# Understanding How Discourse Themes in an Online Mental Health Community on Twitter/X Drive Varied Population-Specific Empowerment Processes in Alignment With Global Standards: A Qualitative Analysis of #BipolarClub

**DOI:** 10.2196/74912

**Published:** 2025-08-29

**Authors:** Horeya AbouWarda, Gianluca Miscione

**Affiliations:** 1 Department of Informatics Faculty of Business, Economics and Informatics University of Zurich Zurich Switzerland; 2 Management Information Systems Group Michael Smurfit Graduate Business School University College Dublin Dublin Ireland; 3 Institute of Business Information Technology, Department of General Management School of Management and Law Zurich University of Applied Sciences Zurich Switzerland

**Keywords:** social media, Twitter/X, online mental health community, OMHC, discourse themes, population-specific empowerment processes, diverse populations, World Health Organization, WHO, Integrated People-Centred Health Services framework (Strategy 1)

## Abstract

**Background:**

Social media, encompassing online mental health communities (OMHCs), has revolutionized mental health discourse by fostering open discussions that drive population-specific empowerment. These discussions generate diverse empowerment processes, such as informational, family awareness, and social awareness support, that can empower different health-consumer populations based on their support needs. However, how OMHC discourse themes drive these empowerment processes remains unexplored.

**Objective:**

This study sought to understand how discourse themes in Twitter/X OMHCs drive three categories of population-specific empowerment processes aligned with all populations in Strategy 1 of the World Health Organization’s (WHO’s) Integrated People-Centred Health Services (IPCHS) framework: (1) individual-level processes for individuals with mental health conditions, including underserved and marginalized individuals (informational, self-expression, network, emotional, esteem, and tangible support); (2) informal carer processes for families and friends (family and friend awareness support); and (3) society-level processes for the broader public (main process: social awareness support). Specifically, it identified the discourse themes, their resonance with OMHC members, and the population-specific empowerment processes they drive.

**Methods:**

We analyzed discussions in the Twitter OMHC #bipolarclub. We collected 2068 tweets using its #bipolarclub hashtag between December 2022 and January 2023, of which 547 were eligible for analysis. We identified the discourse themes using qualitative thematic analysis and defined their resonance with OMHC members based on their prevalence in the tweets. We determined the population-specific empowerment processes driven by these themes by examining the processes embedded within them.

**Results:**

We identified six overarching discourse themes, ranked by prevalence: (1) symptom, medication, treatment, and health care system experiences (187/547, 34.2%); (2) daily life challenges, coping experiences, and recommendations (94/547, 17.2%); (3) socializing and connecting (87/547, 15.9%); (4) mental health awareness and stigma prevention initiatives (69/547, 12.6%); (5) behavioral coaching and motivational dialogue (63/547, 11.5%); and (6) personal feelings, thoughts, experiences, and reflections (47/547, 8.6%). Theme 4’s discussions extended beyond the OMHC’s online environment to involve real-world settings. While all themes generated empowerment processes to different extents across the three population-specific categories, only themes 4 and 5 drove all processes within these categories. Informational support (the most common individual-level process in the discussions) and social awareness support were strongly driven by theme 4, whereas family awareness support and friend awareness support were primarily driven by themes 6 and 2, respectively.

**Conclusions:**

Our analysis highlights the multifaceted roles and ability of Twitter OMHC discourse themes in generating varied population-specific empowerment processes supporting all populations in Strategy 1 (WHO’s IPCHS framework). This demonstrates Twitter-based OMHCs’ potential to foster a comprehensive empowering environment aligned with global standards. This study provides significant insights that can help health care stakeholders develop more tailored OMHC empowering services for diverse populations. Additionally, we present a conceptual framework linking the discourse themes to the three categories of population-specific empowerment processes.

## Introduction

### Background: The Power of Social Media and Online Mental Health Communities in Facilitating Health Care Consumer Empowerment

Social media platforms such as Twitter (recently rebranded as X) [1], Facebook [2], Reddit [3], YouTube [4], Instagram [5], and TikTok [6] have revolutionized the discourse on mental health. These platforms have enabled the creation of online mental health communities (OMHCs), forming dynamic spaces for open discussions that can empower various populations of health care consumers [7]. These consumers include both individuals with health conditions and those in good health, whose involvement with the health care system may be direct (eg, individuals managing health conditions) or indirect (eg, informal carers, family members, and the broader public) [8]. By facilitating such discussions, OMHCs contribute to advancing global empowerment objectives focused on various populations of health care consumers, particularly those outlined in Strategy 1 of the World Health Organization’s (WHO’s) Integrated People-Centred Health Services (IPCHS) framework [8]. To leverage the potential of these online platforms, health care providers—such as health organizations and professionals [1,9,10]—actively engage in these discussions, addressing a wide range of mental health topics aimed at empowering health care consumers. However, their initiatives cannot be fully realized without a clear understanding of how the themes of these discussions drive population-specific empowerment. Lacking this insight may hinder health care providers from effectively tailoring their discussions to meet the specific support needs of different populations. Therefore, such understanding is essential for optimizing health care providers’ engagement with these online platforms to empower diverse populations effectively.

Discussions in OMHCs on social media cover a variety of discourse themes, ranging from mental health advocacy to treatment [3,10-15]. Such discussions allow health care consumers from diverse populations to engage in various consumer “empowerment processes” [7], supporting Strategy 1 of the WHO’s IPCHS framework [8]. These populations include individuals with mental health conditions [16,17], underserved and marginalized individuals with mental health conditions [5,18], informal carers such as family members and friends [3,19], and the broader public (society) [12]. The empowerment processes that these populations could engage in involve varied forms of support, such as informational support, emotional support, self-expression support, network support, esteem support, tangible support, family awareness support, friend awareness support, and social awareness support [7]. Given that these empowerment processes could cater to different populations based on their specific support needs, we define them in this study as *OMHC population-specific empowerment processes*. Participation in these empowerment processes can lead to significant empowerment outcomes for the different populations involved. For example, individuals with mental health conditions may experience improved health self-management, heightened self-esteem and self-efficacy, enhanced navigation of the health care system, and a more active role in health care consultations, which can contribute to better health outcomes and overall life quality [2,20-22]. In addition, informal carers and the broader public can gain a deeper understanding and greater awareness of mental health [7,12].

While extensive research has examined and acknowledged the significant role of OMHC discussions on social media in facilitating diverse population-specific empowerment processes aligned with Strategy 1 of the WHO’s IPCHS framework, how OMHC discourse themes drive these empowerment processes remains unexplored. Thus, to address this knowledge gap and contribute to the existing research and health care practice, this study aimed to provide a deeper understanding of how discourse themes within OMHCs on social media drive varied population-specific empowerment processes consistent with global empowerment standards—particularly Strategy 1 of the WHO’s IPCHS framework [8].

### The Global Movement Toward Health Care Consumer Empowerment: Leveraging Social Media and OMHCs

Consumer empowerment refers to equipping health care consumers both at the individual and population levels with tailored educational resources, support, and opportunities to develop the knowledge, skills, and confidence needed to actively engage in decisions and actions affecting their health and lives [8,23,24]. The consumer empowerment concept is composed of 2 aspects: empowerment processes and empowerment outcomes [7,25-27]. The former refers to activities that have the potential to empower people, whereas the latter represents the result stemming from these processes, indicating the state of being empowered. Consumer empowerment significantly benefits both health care consumers and health care systems. It can enhance health outcomes among consumers as well as improve the effectiveness of the health care system and tackle its critical challenges, such as financial constraints and the shortage of staff [23]. Recognizing these benefits, various health care stakeholders—including health care systems, governmental and nongovernmental organizations (eg, the WHO), professionals, and researchers—have embraced the consumer empowerment notion worldwide [23,28].

**Figure 1:**
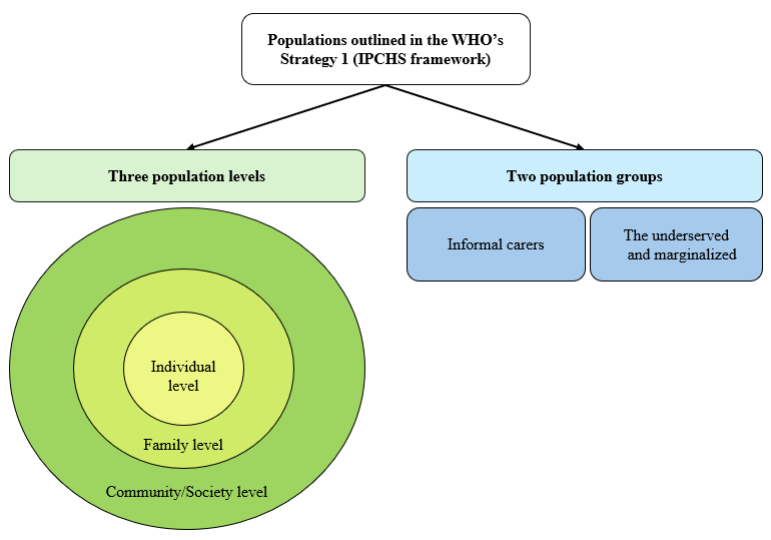
The 3 population levels and the 2 population groups outlined in Strategy 1 of the World Health Organization’s (WHO’s) Integrated People-Centred Health Services (IPCHS) framework. The 3 population levels are illustrated as depicted by the WHO [29].

To guide this global consumer empowerment agenda, the WHO has issued holistic directives through its IPCHS framework, specifically in Strategy 1 (empowering and engaging people and communities) of this framework [8]. This strategy aims to empower diverse populations, as stated in its four substrategies: empowering and engaging individuals and families (Strategy 1.1), empowering and engaging communities (Strategy 1.2), empowering and engaging informal carers (Strategy 1.3), and reaching the underserved and marginalized (Strategy 1.4). While these 4 substrategies contribute to a more comprehensive consumer empowerment notion in health care, each substrategy offers a distinct perspective tailored to a specific population level(s) (individual level, family level, and community/society level) or population group (informal carers and the underserved and marginalized). These populations are represented in Figure 1.

In addition, focusing on mental health, the WHO has affirmed Strategy 1’s directives in various statements, such as the Comprehensive Mental Health Action Plan 2013-2030 [30], the World Mental Health Report [31], and the Guidance on Community Mental Health Services [32]. In these 3 statements, the WHO has actively urged related institutions and experts, including academic and research entities [30], to expand and enhance the integration of social media and online communities as digital mental health tools in the health care system. Furthermore, the IPCHS framework [8], with Strategy 1 as part of it, has called “...for reforms to reorient health services, putting individuals, families, carers and communities at their centre, supported by responsive services that better meet their needs, and that are coordinated both within and beyond the health sector...” This statement highlights the need for more inclusive and accessible health care services that extend beyond traditional health system infrastructures.

OMHCs on social media play a pivotal role in fostering a more inclusive and accessible consumer empowerment approach in mental health care. These communities serve as open and accessible platforms where health care consumers from diverse populations and various health care stakeholders (eg, health organizations and professionals) can seamlessly interact and exchange knowledge and insights [1,5,10-12,16-19]. Such knowledge cocreation interactions could not only address the specific needs of health care consumers within these online communities but also improve the effectiveness and responsiveness of health care services in both online and offline settings [33-35]. For example, these interactions can inform the development of consumer empowerment services customized for diverse populations in OMHCs while also contributing to the enhancement of traditional mental health services. Therefore, OMHCs emerge as promising tools for achieving the goals of the WHO’s IPCHS framework and its Strategy 1, offering a more comprehensive and accessible model of consumer empowerment that expands beyond conventional health care approaches.

Despite the active use of OMHCs on social media by various health care stakeholders—such as health organizations and experts [1,9,10]—who engage in mental health discussions to empower health care consumers, their efforts remain limited. These efforts cannot be fully achieved without a clear understanding of how the themes of these discussions generate population-specific empowerment processes for diverse populations. Specifically, it is essential to determine what discourse themes are commonly discussed in these online communities and how these themes are linked to the generation of diverse population-specific empowerment processes. This understanding is crucial for those health care stakeholders (eg, health organizations, professionals, and OMHC moderators) who aim to fully harness the potential of OMHCs. It can help them generate more tailored, targeted, and impactful empowering discussions that address the specific empowerment needs of different populations. For example, if a mental health organization seeks to empower a particular population—such as individuals with mental health conditions, their families and friends, or the broader public—it can strategically organize discussions on discourse themes that stimulate empowerment processes relevant to this population within OMHCs. However, without this understanding, the mental health organization may struggle to identify and coordinate the appropriate discourse themes that could effectively drive the desired empowerment processes for the target population. In addition, several studies have emphasized the need for deeper insights into the practices carried out within OMHCs on social media and their role in promoting consumer empowerment [2,20]. Furthermore, gaining a profound understanding of OMHCs is especially important given their widespread use and the fact that mental health conditions are recognized as the leading causes of disability worldwide [10], with their prevalence continuing to rise [36]. This highlights the significance of acquiring valuable insights into these online communities to fully harness their potential and enrich the existing body of research in this domain.

### Discourse Themes and Consumer Empowerment Processes in OMHC Discussions on Social Media

Several studies have investigated discourse themes and consumer empowerment processes in OMHC discussions on social media. For example, some studies have explored the discourse themes, identifying various topics related to symptoms, diagnosis, medication, coping mechanisms, recovery, daily and social struggles associated with mental health conditions, experiences with mental health services, and mental health literacy and stigma [3,6,12-15,37-40]. These discourse themes not only are of interest to individuals directly experiencing mental health conditions but also resonate with other populations of health care consumers (eg, informal carers and the broader public). For instance, a study focusing on Reddit’s online communities has shown that some youths engaged in discussions to seek advice on how to help their friends or partners who deal with mental health challenges [3]. In addition, previous research has reported that mental health treatment has emerged as a common theme of discussion on social media [40]. These studies could suggest that OMHCs on social media play a critical role in addressing gaps in traditional health care services, such as the limited availability of mental health professionals, inadequate insurance coverage for mental health services, insufficient support for racial and ethnic minority populations (eg, Black population), and a lack of services for informal carers [3,17-19,41]. A preference among health care consumers for engaging in such discussions on these online platforms, where personal experiences—resources that may not be readily accessible elsewhere—are shared [6], further underscores the value of OMHCs on social media in bridging these gaps. By providing a space for open dialogue, these platforms improve access to mental health resources and services that might otherwise be unavailable or difficult to obtain.

Although OMHCs on social media offer significant benefits, they are not exempt from the negative aspects inherent to online environments. A number of studies have highlighted some risks associated with discussions on these platforms, such as the promotion of harmful behaviors within online pro–eating disorder communities [42,43], as well as the romanticization of mental health conditions that can foster inaccurate beliefs about these conditions [43]. The dissemination of inaccurate health-related information within such discussions is another concern as it can lead to harmful consequences [44,45]. Despite these drawbacks, the pivotal role of OMHCs in advancing consumer empowerment across various populations remains undeniable due to their capability to enhance access to mental health resources and resolve current service deficiencies.

Some studies have also examined consumer empowerment processes (empowering activities) within OMHC discussions, highlighting how these discussions encompass diverse empowerment processes that address the specific support needs of different populations. For instance, a recent study focusing on discussions within a Twitter-based OMHC identified 3 categories of consumer empowerment processes [7]. These 3 categories correspond to all populations outlined in Strategy 1 of the WHO’s IPCHS framework. They involved (1) individual-level empowerment processes for individuals with mental health conditions, including underserved and marginalized individuals (informational support, self-expression support, network support, emotional support, esteem support, and tangible support); (2) informal carer empowerment processes for family members and friends (family awareness support and friend awareness support); and (3) society-level empowerment processes for the broader public (main process: social awareness support). This study, which addressed all diverse populations in the WHO’s Strategy 1, has demonstrated that OMHC discussions include what we define in the current study as *OMHC population-specific empowerment processes*. Other studies have also investigated consumer empowerment processes in OMHC discussions but have often focused on a specific population, such as individuals with mental health conditions [17,22,46], underserved and marginalized individuals [5,18], and informal carers such as family members [19]. All of those studies reveal that OMHC discussions encompass diverse population-specific empowerment processes consistent with Strategy 1 of the WHO’s IPCHS framework.

As clarified, previous studies have extensively explored discourse themes and consumer empowerment processes in OMHC discussions, recognizing that these discussions involve various themes and diverse population-specific empowerment processes aligned with Strategy 1 of the WHO’s IPCHS framework. However, they have primarily focused on themes and empowerment processes separately or combined (eg, framing empowerment processes as part of discourse themes), overlooking how different themes may drive varied population-specific empowerment processes in OMHC discussions. Given the WHO’s call to leverage social media and online communities as digital tools in mental health care [30-32], it is crucial to deepen our understanding of how discourse themes in OMHC discussions generate varied population-specific empowerment processes corresponding to the WHO’s Strategy 1. This knowledge is essential for guiding the effective use of OMHCs. Moreover, focusing on global consumer empowerment standards for diverse populations (WHO’s Strategy 1) represents an important area of research [47] as it can support global initiatives aimed at addressing mental health inequalities and improving access to care worldwide.

### Aims of This Study

Therefore, this study sought to provide a deeper understanding of and insights into how discourse themes within Twitter-based OMHCs drive varied population-specific empowerment processes aligned with Strategy 1 of the WHO’s IPCHS framework [7]. We aimed to identify the discourse themes in these communities, define the extent to which these themes resonate with OMHC members, and specify the population-specific empowerment processes generated by these themes. By concentrating on discourse themes in OMHC discussions, we intended to determine the OMHC population-specific empowerment processes that are enabled by these themes.

We chose to focus on OMHCs on Twitter, seeking to maximize the advantages of social media and OMHCs in advancing global empowerment endeavors across various populations. Twitter is among the most widely used social media platforms in health care activities [48-50]. It serves as a publicly accessible platform where users can participate in empowering discussions through user-friendly content dissemination features, fostering widespread distribution of content to a broader audience without limitations associated with account or community followership [51,52]. These features could foster the creation of a mental health support ecosystem able to reach a wide audience, underscoring its potential to support global empowerment initiatives for various populations in line with Strategy 1 of the WHO’s IPCHS framework. Online communities on Twitter often emerge organically from the ground up, revolving around particular hashtags. These hashtags, which begin with a “#” sign followed by a word or more associated with a specific subject, serve as identifiers for tweets. These hashtags enable multiple users to access and contribute to discussions about the subject, shaping communities of users who engage in conversations around common interests, exemplified by hashtags such as #depression and #schizophrenia [53], #depressionsucks [13], #MyDepressionLooksLike [37], #WhyWeTweetMH [54], and #dearmentalhealthprofessionals [55].

To guide our inquiry, we developed two research questions (RQs) for investigating the Twitter-based OMHC known as #bipolarclub:

- RQ 1: What are the discourse themes in the OMHC’s discussions, and to what extent do these themes resonate with its members?
- RQ 2: What are the OMHC population-specific empowerment processes driven by these discourse themes in line with the following three categories of population-specific processes corresponding to the populations stated in Strategy 1 of the WHO’s IPCHS framework? (1) individual-level processes for individuals coping with mental health conditions, including those from underserved and marginalized populations; (2) informal carer processes for family members and friends; (3) society-level processes for the broader public.

## Methods

### Overview

This study expands upon the findings reported in “How Does an Online Mental Health Community on Twitter Empower Diverse Population Levels and Groups? A Qualitative Analysis of #BipolarClub,” which discussed the types of empowerment processes aligned with the population levels and groups outlined in Strategy 1 of the WHO’s IPCHS framework within the #bipolarclub community [7]. Initially, the plan was to carry out a comprehensive investigation on the consumer empowerment notion within the #bipolarclub community considering both the empowerment processes and the discourse themes. However, due to the richness and volume of information, a decision was made to split the findings into 2 separate studies, each focusing on a distinct but interrelated aspect of the data and their analysis (the previous related study on the empowerment processes [7] and the current one on the discourse themes).

By using the same data and building on previous findings (specifically, the previously analyzed empowerment processes [7]), this approach allowed for a deeper interpretation and analysis in the current study, particularly the connection between the discourse themes and the empowerment processes, thereby strengthening its rigor through more nuanced insights and findings. In addition, the current study is part of a broader ongoing investigation into consumer empowerment in the #bipolarclub community using a netnographic methodology [56,57]. This approach relies on immersive longitudinal analysis of behaviors within online communities. Applying this methodology has supported the continued relevance of our findings despite the earlier timing of the data analysis, which aligns with the previously published related study [7], as the broader investigation has remained active and the online community has continued to be observed.

### Research Setting and Data Collection

To address our research objectives, we conducted this study on the Twitter-based OMHC #bipolarclub. The community’s Twitter account, through which its moderators communicate with members, is named “@BipolarClubDx” [58]. As clarified on its website [59], this OMHC was created in March 2021 by individuals navigating mental health conditions.

For several reasons, this community was chosen as a relevant and ideal setting for our research context, offering valuable insights and opportunities for exploration. First, it is an inclusive platform that extends beyond bipolar disorder and those coping with this condition, aligning with our research focus on empowerment for diverse populations. Although its name may imply a primary emphasis on bipolar disorder and a main objective of supporting individuals facing it, a pinned tweet on the community’s Twitter account explicitly indicates that this OMHC is not restricted to a particular disorder or group of people [58]. Instead, it encompasses a wider scope, addressing mental health issues in general and welcoming anybody interested in the conversation. Second, the #bipolarclub community aligns with our research focus as a well-established entity on Twitter rather than merely a trendy mental health hashtag. It is more than just a gathering around a particular hashtag; it is a well-structured OMHC with a dedicated Twitter account maintained by moderators responsible for its management. These features distinguish it as a stable and lasting online community rather than a temporary trend-driven entity. Lastly, the #bipolarclub community is characterized by its active, diverse, and rich interactions, making it an ideal candidate for exploratory research on online communities [57,60].

The community uses 2 primary communication channels on Twitter, represented by the hashtags #bipolarclub and #DBTclub [61], as well as public audio conversations hosted by the community’s Twitter account on Twitter Spaces [62]. These Twitter Spaces are managed by the #bipolarclub community’s moderators and center on the topics related to these 2 hashtags. The hashtag #bipolarclub serves as the main hashtag for members to discuss a wide range of topics within the OMHC, whereas #DBTclub is specifically used for discussions related to dialectical behavior therapy (DBT). Both hashtags are also used to share announcements related to the audio conversations on Twitter Spaces, such as upcoming sessions or session recordings. In alignment with the hashtags’ themes, the audio conversations act as online mental health support and awareness sessions, where broader mental health topics are covered under #bipolarclub and DBT-focused discussions take place under #DBTclub. Notably, while #DBTclub and its associated Twitter Spaces have distinct purposes, any posts using the hashtag #DBTclub integrate the main hashtag #bipolarclub of the community, making DBT-related discussions a part of the larger community conversation.

We used the Twitter search application programming interface (API) [63] in conjunction with the Postman API development environment (Postman Inc) to collect tweets involving the #bipolarclub hashtag from Twitter. In total, we gathered 2068 tweets posted by members of the #bipolarclub community between December 19, 2022, and January 15, 2023. While our analysis considered both of the community’s hashtags, #bipolarclub and #DBTclub, we specifically collected tweets containing #bipolarclub as the #DBTclub hashtag is inherently included in those tweets. The data collected for each tweet included its content and its author’s Twitter profile details. From the initial 2068 tweets retrieved, after filtering out retweets and quote tweets (n=1105, 53.4%), non–English-language tweets (n=243, 11.8%), and those lacking text or with irrelevant content (n=173, 8.4%), our final dataset included 547 (26.5%) tweets. While our primary focus was on the textual content of tweets, we also incorporated media elements, including emojis, images, videos, Graphics Interchange Format (GIF) images, and external links, into our analysis.

### Ethical Considerations

This study carefully considered several key ethical aspects related to the use of social media data. First, discussions within the #bipolarclub community on Twitter were publicly accessible as only tweets from users with public profiles could be retrieved, ensuring that private accounts were automatically excluded from the research. The availability of these public tweets permits their content to be used for research purposes [12], which also aligns with the requirements for waiving informed consent [64,65]. Second, before commencing the study, the first author (HA) ethically communicated the research objectives to the #bipolarclub community’s crew through private Twitter messages, resulting in their support for the study. Third, to ensure the privacy and confidentiality of #bipolarclub community members [66], any identifiable information (eg, @usernames and names) in the reported tweets was substituted with pseudonyms after the analysis, excluding those related to mental health care institutions. In addition, the tweets were paraphrased to preserve anonymity. This research was approved by the Human Subjects Committee of the Faculty of Business, Economics and Informatics at the University of Zurich (OEC IRB #2022-093).

### Qualitative Analysis

We applied qualitative content analysis to examine and interpret the data [67] using NVivo software (QSR International). We used this analytical approach because it has been used and substantiated in numerous previous studies for analyzing social media interactions and content [3,40]. Qualitative analysis allows for a deep understanding of behavioral patterns that extend beyond what purely statistical methods can typically reveal. While its primary focus is interpretive, it can also incorporate quantitative elements such as frequency counts to support and contextualize its findings [54,67,68].

To identify the discourse themes within the #bipolarclub community, we used inductive thematic analysis [68], which allows themes to emerge directly from the data. The analysis was conducted in accordance with the steps defined by Braun and Clarke [68] for thematic analysis: (1) familiarizing oneself with the data, (2) generating initial codes, (3) searching for themes, (4) reviewing themes, and (5) defining and naming themes. The first author (HA) began by repeatedly reading the tweets to become familiar with the data, generating initial codes that reflected the content. Next, HA organized these codes into potential themes and subthemes, ensuring that they accurately represented the data. The preliminary themes were then reviewed and refined through discussions with the second author (GM), ultimately leading to the identification of robust themes that encapsulated the discourse within the #bipolarclub community. We defined the resonance of these themes with the community’s members based on their prevalence within the tweets.

As part of a broader study on consumer empowerment within the #bipolarclub community, the current study examined how the emerged discourse themes relate to generating the 3 categories of OMHC population-specific empowerment processes aligned with Strategy 1 of the WHO’s IPCHS framework [7]. A detailed analysis of how these empowerment processes were identified within the tweets was discussed separately in the previous study related to this one [7] as it extends beyond the scope of the current study. Here, we built on that previous study [7] and focused specifically on analyzing these identified empowerment processes in relation to the emerging discourse themes and subthemes reflected in the tweets. The three identified categories of OMHC population-specific empowerment processes were as follows [7]: (1) individual-level empowerment processes (relevant to individuals with mental health conditions, including underserved and marginalized individuals); (2) informal carer empowerment processes (targeted at family members and friends); and (3) society-level empowerment processes (aimed at the broader public). The individual-level category included 6 processes and 2 subprocesses: informational support (experiential informational support and objective informational support), self-expression support, network support, emotional support, esteem support, and tangible support. The informal carer category involved 2 processes: family awareness support and friend awareness support. The society-level category incorporated 1 process and 2 subprocesses: social awareness support (which encompassed perceptive awareness support and destigmatization support). Table 1 provides a detailed description of these 3 categories and their corresponding empowerment processes (the processes within each category are listed from top to bottom in descending order of prevalence in the #bipolarclub community [7]).

Each tweet was coded using two coding schemes: (1) the most relevant discourse theme and subtheme and (2) all relevant consumer empowerment processes and subprocesses across the 3 population-specific categories. This coding approach allowed us to link the discourse themes and subthemes to the specific empowerment processes that they drove through the tweets within the #bipolarclub community. Each tweet was assigned to only 1 most relevant discourse theme to avoid overlap and ensure that the primary focus of the tweet’s theme was clearly identified. In contrast, all relevant population-specific empowerment processes were coded in each tweet, as a single tweet could simultaneously reflect multiple perspectives of empowerment.

**Table 1:** The 3 categories of OMHC population-specific empowerment processes. OMHC: online mental health community.

Category of population-specific empowerment processes and relevant process and subprocess				Characterization
**Individual-level empowerment processes**				Empowerment processes for individuals with mental health conditions, including individuals from underserved and marginalized populations (addressing the specific needs of individuals from underserved and marginalized populations, including children and individuals from racial and ethnic minority groups [eg, Black population]).
	**Informational support**		Provision of health-related information about mental health conditions and coping strategies, including both objective and fact-based information as well as experiential insights derived from personal experiences.	
		Experiential informational support	Provision of information derived from personal experiences, offering firsthand insights into navigating everyday life with mental health conditions as well as self-management and coping mechanisms, including medical, therapeutic, and health care system experiences.	
		Objective informational support	Provision of information that is impartial and fact based, including mental health–related education materials, advice, and referrals.	
	**Self-expression support**		Self-disclosing personal feelings, thoughts, daily life experiences, and challenges of living with mental health conditions, as well as self-motivational expressions.	
	**Network support**		Communicating with affiliation to the online community as well as provision of offers to gain access to its members.	
	**Emotional support**		Provision of care, love, encouragement, and understanding expressions.	
	**Esteem support**		Provision of affirmation in ability and compliment expressions, as well as expressions of agreement on a situation and alleviating a sense of guilt about a situation.	
	**Tangible support**		Provision of offers to help and to join activities or events that are needed to cope with the challenges of mental health conditions, including online and offline peer support groups.	
**Informal carer empowerment processes**				Empowerment processes for family members and friends.
	**Family awareness support**		Provision of information that pertains to both mental health– and family-related aspects, including personal experiences with families.	
	**Friend awareness support**		Provision of information that pertains to both mental health– and friend-related aspects, including personal experiences with friends.	
**Society-level empowerment processes**				Empowerment processes for the broader public.
	**Social awareness support**		Provision of information that pertains to both mental health– and societal-related aspects, addressing social misconceptions and stigmatization of mental health conditions and offering perspectives on the actual experiences and realities associated with mental health conditions.	
		Perceptive awareness support	Provision of information that addresses social misconceptions surrounding mental health conditions, including clarifications of these misconceptions, insights into the reality of mental health conditions, and personal experiences in society.	
		Destigmatization support	Provision of information that addresses the destigmatization of mental health conditions in society, including antistigma expressions and personal experiences with mental health stigma.	

## Results

### Overall Results on Discourse Themes in OMHC Discussions and Population-Specific Empowerment Processes Driven by OMHC Discourse Themes

From a total of 547 #bipolarclub tweets, 6 overarching discourse themes and 22 associated subthemes emerged. These six themes, ranked by prevalence from most to least common within the #bipolarclub community, were (1) symptom, medication, treatment, and health care system experiences; (2) daily life challenges, coping experiences, and recommendations; (3) socializing and connecting; (4) mental health awareness and stigma prevention initiatives; (5) behavioral coaching and motivational dialogue; and (6) personal feelings, thoughts, experiences, and reflections. The discourse themes were shaped by different types of discussions within the community, including self-discussions (eg, self-motivation, self-disclosure, and venting), interpersonal discussions (with other community members), and spiritual discussions (eg, praying).

**Figure 2:**
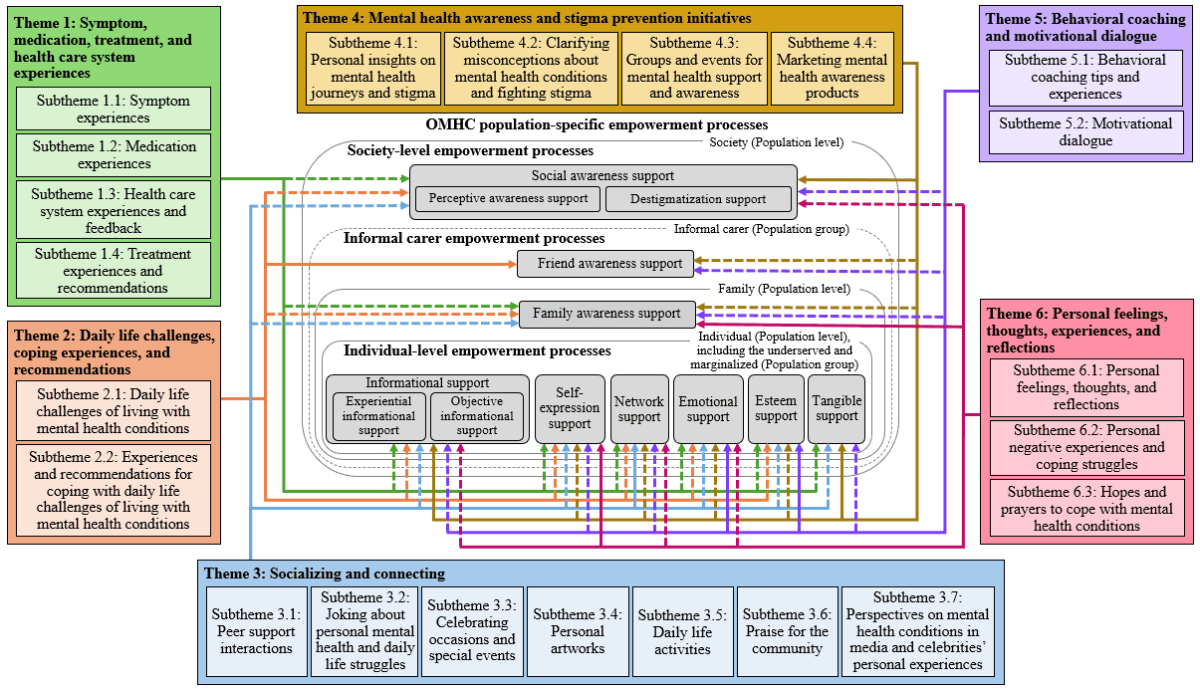
A conceptual framework illustrating how the #bipolarclub community’s discourse themes drive the 3 categories of OMHC population-specific empowerment processes aligned with the populations noted in Strategy 1 of the World Health Organization’s Integrated People-Centred Health Services framework. The 3 categories of population-specific empowerment processes are structured according to the 3 population levels and the 2 population groups outlined in the World Health Organization’s Strategy 1, which these processes could empower (this structure is adapted from the study by AbouWarda et al [7]). Each arrow, matched in color to its corresponding discourse theme, connects the theme to the population-specific empowerment process it drives. A solid arrow indicates that the discourse theme strongly drives the empowerment process. OMHC: online mental health community.

**Figure 3:**
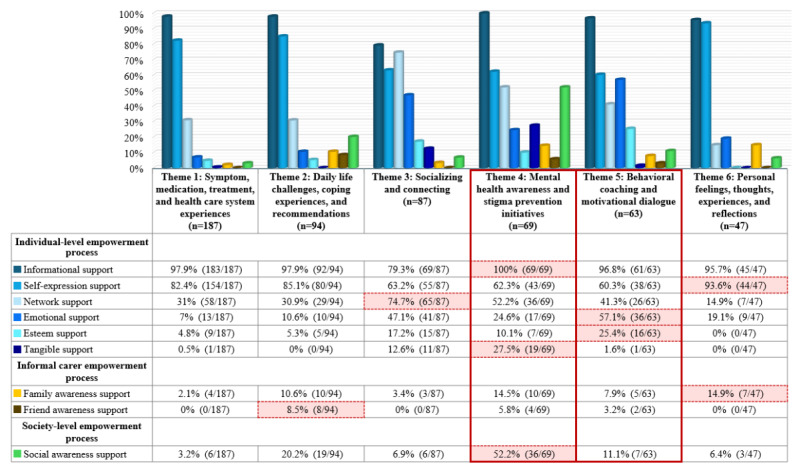
An overview of proportions for the 3 categories of OMHC population-specific empowerment processes within the discourse themes of tweets (N=547) in the #bipolarclub community. The discourse themes are arranged horizontally from the most to the least discussed within the #bipolarclub community, whereas the population-specific empowerment processes are organized vertically from the most to the least common within the online community according to each of the 3 categories. The population-specific empowerment processes strongly driven by the discourse themes are highlighted with red-shaded dotted boxes. The only 2 themes that drove all the empowerment processes across the 3 categories are marked with red solid boxes. OMHC: online mental health community.

Overall, as shown in Figures 2 and 3, all 6 discourse themes, through their related tweets, generated empowerment processes to varying degrees (Figure 3) across all three categories of population-specific processes: (1) individual-level empowerment processes supporting individuals with mental health conditions, including those from underserved and marginalized populations; (2) informal carer empowerment processes, supporting family members and friends; and (3) society-level empowerment processes, supporting the broader public. This finding suggests that all the discourse themes in the #bipolarclub community could advance empowerment across diverse populations—particularly those outlined in Strategy 1 of the WHO’s IPCHS framework. By addressing health-related, personal, and social aspects, these themes could help the community members navigate their mental health journey and promote collective mental health awareness. Figure 2 shows a conceptual framework illustrating how the identified discourse themes in the #bipolarclub community drive the 3 categories of population-specific empowerment processes, and Figure 3 provides an overview of the proportions of these processes within the themes.

While the 6 identified discourse themes enabled empowerment processes to different extents across the 3 categories of population-specific processes, our analysis revealed that only themes 4 and 5 drove all the empowerment processes within these 3 categories (Figure 3). This finding highlights the crucial role of themes 4 (mental health awareness and stigma prevention initiatives) and 5 (behavioral coaching and motivational dialogue) in comprehensively supporting empowerment across diverse populations.

In addition, as illustrated in Figure 3 (with detailed percentages) and Figure 2 (providing an overview), our examination demonstrated that different discourse themes strongly drove the population-specific empowerment processes across the 3 categories, highlighting the influential role of the themes in shaping the type of empowerment generated in the #bipolarclub community. For example, we found that informational support (the most prevalent individual-level empowerment process within the discourse themes in this study and consistent with previous findings in the #bipolarclub community [7]) and social awareness support (the main society-level empowerment process) were strongly driven by theme 4 (mental health awareness and stigma prevention initiatives). Meanwhile, family awareness support and friend awareness support (the 2 informal carer empowerment processes) were primarily driven by themes 6 (personal feelings, thoughts, experiences, and reflections) and 2 (daily life challenges, coping experiences, and recommendations), respectively. Table 2 presents detailed frequencies of the discourse themes and subthemes in the tweets, as well as the proportions of the empowerment processes across the 3 categories of population-specific processes in each theme and subtheme. For further details, Multimedia Appendix 1 provides sample paraphrased tweets for each identified discourse theme and subtheme, along with the population-specific empowerment processes involved in each tweet. Unlike the previous related study [7], which included excerpts of tweets to illustrate the empowerment processes, the current study presents nearly complete tweets that are mostly different from those previously reported [7] and were carefully selected to comprehensively represent both the discourse themes and subthemes and the associated empowerment processes. Some samples of these tweets are included in the following Results subsections to illustrate key findings, whereas others are exclusively presented in Multimedia Appendix 1.

**Table 2:** Discourse themes and subthemes derived from the 547 #bipolarclub tweets, their frequencies in the tweets, and the proportions of the OMHC population-specific empowerment processes of the 3 categories (individual-level processes, informal carer processes, and society-level processes) in each discourse theme and subtheme. OMHC: online mental health community.

Theme and subtheme			Tweet frequency, n (%)		Proportions of OMHC population-specific empowerment processes, n (%)				
					Individual-level empowerment processes		Informal carer empowerment processes		Society-level empowerment processes
**Symptom, medication, treatment, and health care system experiences**			187 (34.2)		IS^a^: 183 (97.9); 180 (96.3) EIS^b^ and 23 (12.3) OIS^c^ SS^d^: 154 (82.4) NS^e^: 58 (31) EmS^f^: 13 (7) EsS^g^: 9 (4.8) TS^h^: 1 (0.5)		FaAS^i^: 4 (2.1)		SAS^j^: 6 (3.2); 6 (3.2) PAS^k^ and 1 (0.5) DS^l^
	Symptom experiences	77 (14.1)		IS: 77 (100); 76 (98.7) EIS and 3 (3.9) OIS SS: 71 (92.2) NS: 20 (26) EmS: 2 (2.6) EsS: 2 (2.6)		—^m^		SAS: 2 (2.6); 2 (2.6) PAS	
	Medication experiences	53 (9.7)		IS: 50 (94.3); 49 (92.5) EIS and 6 (11.3) OIS SS: 44 (83) NS: 17 (32.1) EmS: 3 (5.7) EsS: 2 (3.8)		FaAS: 2 (3.8)		SAS: 3 (5.7); 3 (5.7) PAS	
	Health care system experiences and feedback	35 (6.4)		IS: 35 (100); 35 (100) EIS and 5 (14.3) OIS SS: 28 (80) NS: 10 (28.6) EmS: 8 (22.9) EsS: 5 (14.3) TS: 1 (2.9)		FaAS: 1 (2.9)		SAS: 1 (2.9); 1 (2.9) PAS and 1 (2.9) DS	
	Treatment experiences and recommendations	22 (4)		IS: 21 (95.5); 20 (90.9) EIS and 9 (40.9) OIS SS: 11 (50) NS: 11 (50)		FaAS: 1 (4.5)		—	
**Daily life challenges, coping experiences, and recommendations**			94 (17.2)		IS: 92 (97.9); 88 (93.6) EIS and 10 (10.6) OIS SS: 80 (85.1) NS: 29 (30.9) EmS: 10 (10.6) EsS: 5 (5.3)		FaAS: 10 (10.6) FrAS^n^: 8 (8.5)		SAS: 19 (20.2); 19 (20.2) PAS
	Daily life challenges of living with mental health conditions	73 (13.3)		IS: 73 (100); 71 (97.3) EIS and 3 (4.1) OIS SS: 72 (98.6) NS: 18 (24.7) EmS: 4 (5.5) EsS: 1 (1.4)		FaAS: 6 (8.2) FrAS: 4 (5.5)		SAS: 17 (23.3); 17 (23.3) PAS	
	Experiences and recommendations for coping with daily life challenges of living with mental health conditions	21 (3.8)		IS: 19 (90.5); 17 (81) EIS and 7 (33.3) OIS NS: 11 (52.4) SS: 8 (38.1) EmS: 6 (28.6) EsS: 4 (19)		FaAS: 4 (19) FrAS: 4 (19)		SAS: 2 (9.5); 2 (9.5) PAS	
**Socializing and connecting**			87 (15.9)		IS: 69 (79.3); 61 (70.1) EIS and 18 (20.7) OIS NS: 65 (74.7) SS: 55 (63.2) EmS: 41 (47.1) EsS: 15 (17.2) TS: 11 (12.6)		FaAS: 3 (3.4)		SAS: 6 (6.9); 6 (6.9) PAS
	Peer support interactions	27 (4.9)		NS: 26 (96.3) IS: 22 (81.5); 21 (77.8) EIS and 6 (22.2) OIS SS: 15 (55.6) EmS: 13 (48.1) TS: 8 (29.6) EsS: 6 (22.2)		FaAS: 1 (3.7)		SAS: 1 (3.7); 1 (3.7) PAS	
	Joking about personal mental health and daily life struggles	23 (4.2)		IS: 23 (100); 22 (95.7) EIS and 1 (4.3) OIS SS: 20 (87) NS: 10 (43.5) EmS: 3 (13) EsS: 1 (4.3)		FaAS: 1 (4.3)		SAS: 4 (17.4); 4 (17.4) PAS	
	Celebrating occasions and special events	21 (3.8)		EmS: 19 (90.5) NS: 18 (85.7) IS: 13 (61.9); 10 (47.6) EIS and 4 (19) OIS SS: 9 (42.9) EsS: 4 (19) TS: 3 (14.3)		—		—	
	Personal artworks	5 (0.9)		IS: 4 (80); 4 (80) EIS and 2 (40) OIS SS: 4 (80) EmS: 2 (40) NS: 1 (20)		—		SAS: 1 (20); 1 (20) PAS	
	Daily life activities	4 (0.7)		IS: 3 (75); 2 (50) OIS and 1 (25) EIS SS: 3 (75) NS: 3 (75) EmS: 1 (25) EsS: 1 (25)		FaAS: 1 (25)		—	
	Praise for the community	4 (0.7)		NS: 4 (100) EmS: 3 (75) EsS: 3 (75) IS: 2 (50); 2 (50) EIS and 1 (25) OIS SS: 2 (50)		—		—	
	Perspectives on mental health conditions in media and celebrities’ personal experiences	3 (0.5)		NS: 3 (100) IS: 2 (66.7); 2 (66.7) OIS and 1 (33.3) EIS SS: 2 (66.7)		—		—	

**Mental health awareness and stigma prevention initiatives**			69 (12.6)		IS: 69 (100); 60 (87) EIS and 31 (44.9) OIS SS: 43 (62.3) NS: 36 (52.2) TS: 19 (27.5) EmS: 17 (24.6) EsS: 7 (10.1)		FaAS: 10 (14.5) FrAS: 4 (5.8)		SAS: 36 (52.2); 25 (36.2) PAS and 15 (21.7) DS

	Personal insights on mental health journeys and stigma	32 (5.9)		IS: 32 (100); 32 (100) EIS and 10 (31.2) OIS SS: 26 (81.2) EmS: 12 (37.5) NS: 11 (34.4) TS: 8 (25) EsS: 5 (15.6)		FaAS: 9 (28.1) FrAS: 3 (9.4)		SAS: 17 (53.1); 12 (37.5) DS and 8 (25) PAS	
	Clarifying misconceptions about mental health conditions and fighting stigma	20 (3.7)		IS: 20 (100); 18 (90) EIS and 4 (20) OIS SS: 17 (85) NS: 9 (45) EmS: 5 (25) EsS: 2 (10) TS: 2 (10)		FaAS: 1 (5) FrAS: 1 (5)		SAS: 16 (80); 14 (70) PAS and 3 (15) DS	
	Groups and events for mental health support and awareness	15 (2.7)		IS: 15 (100); 15 (100) OIS and 9 (60) EIS NS: 15 (100) TS: 9 (60)		—		SAS: 1 (6.7); 1 (6.7) PAS	
	Marketing mental health awareness products	2 (0.4)		IS: 2 (100); 2 (100) OIS and 1 (50) EIS NS: 1 (50)		—		SAS: 2 (100); 2 (100) PAS	
**Behavioral coaching and motivational dialogue**			63 (11.5)		IS: 61 (96.8); 45 (71.4) EIS and 19 (30.2) OIS SS: 38 (60.3) EmS: 36 (57.1) NS: 26 (41.3) EsS: 16 (25.4) TS: 1 (1.6)		FaAS: 5 (7.9) FrAS: 2 (3.2)		SAS: 7 (11.1); 6 (9.5) PAS and 1 (1.6) DS
	Behavioral coaching tips and experiences	40 (7.3)		IS: 40 (100); 24 (60) EIS and 18 (45) OIS NS: 21 (52.5) SS: 16 (40) EmS: 13 (32.5) EsS: 5 (12.5) TS: 1 (2.5)		FaAS: 4 (10) FrAS: 1 (2.5)		SAS: 6 (15); 6 (15) PAS	
	Motivational dialogue	23 (4.2)		EmS: 23 (100) SS: 22 (95.7) IS: 21 (91.3); 21 (91.3) EIS and 1 (4.3) OIS EsS: 11 (47.8) NS: 5 (21.7)		FaAS: 1 (4.3) FrAS: 1 (4.3)		SAS: 1 (4.3); 1 (4.3) DS	
**Personal feelings, thoughts, experiences, and reflections**			47 (8.6)		IS: 45 (95.7); 34 (72.3) EIS and 12 (25.5) OIS SS: 44 (93.6) EmS: 9 (19.1) NS: 7 (14.9)		FaAS: 7 (14.9)		SAS: 3 (6.4); 3 (6.4) PAS and 1 (2.1) DS
	Personal feelings, thoughts, and reflections	30 (5.5)		IS: 30 (100); 20 (66.7) EIS and 10 (33.3) OIS SS: 29 (96.7) NS: 4 (13.3) EmS: 3 (10)		—		SAS: 3 (10); 3 (10) PAS and 1 (3.3) DS	

	Personal negative experiences and coping struggles	9 (1.6)		IS: 8 (88.9); 8 (88.9) EIS and 1 (11.1) OIS SS: 7 (77.8) NS: 3 (33.3) EmS: 1 (11.1)		FaAS: 7 (77.8)		—	
	Hopes and prayers to cope with mental health conditions	8 (1.5)		SS: 8 (100) IS: 7 (87.5); 6 (75) EIS and 1 (12.5) OIS EmS: 5 (62.5)		—		—	

^a^IS: informational support.

^b^EIS: experiential informational support.

^c^OIS: objective informational support.

^d^SS: self-expression support.

^e^NS: network support.

^f^EmS: emotional support.

^g^EsS: esteem support.

^h^TS: tangible support.

^i^FaAS: family awareness support.

^j^SAS: social awareness support.

^k^PAS: perceptive awareness support.

^l^DS: destigmatization support.

^m^Not applicable.

^n^FrAS: friend awareness support.

### Theme 1: Symptom, Medication, Treatment, and Health Care System Experiences

#### Overview

The most common discourse theme that emerged from the #bipolarclub community’s tweets centered on exchanging personal experiences with symptoms, medications, treatments, and dealing with health care systems, along with related recommendations. In total, this theme included 34.2% (187/547) of the tweets. We identified four subthemes of discussions within this theme, listed from most to least prevalent: (1) symptom experiences (77/547, 14.1%), (2) medication experiences (53/547, 9.7%), (3) health care system experiences and feedback (35/547, 6.4%), and (4) treatment experiences and recommendations (22/547, 4%).

In total, tweets within this discourse theme (187/547, 34.2%) drove all 6 individual-level empowerment processes, primarily facilitating informational support (183/187, 97.9%), with experiential informational support (180/187, 96.3%) having the highest prevalence rate in this theme compared to other themes, followed by self-expression support (154/187, 82.4%), network support (58/187, 31%), emotional support (13/187, 7%), esteem support (9/187, 4.8%), and tangible support (1/187, 0.5%). It also facilitated 1 of the 2 informal carer empowerment processes, specifically family awareness support, occurring in 2.1% (4/187) of the tweets, and the society-level empowerment process, social awareness support, appearing in 3.2% (6/187) of the tweets.

#### Subtheme 1.1: Symptom Experiences

Many #bipolarclub community members tweeted about their personal experiences with mental disorder symptoms (77/547, 14.1%). Some members shared their struggles with symptoms of these conditions, whereas others sought information from those who might be experiencing similar symptoms to better understand their own conditions:

Feeling the depression returning. I think I should feel thankful this time that it didn’t drop as usual like A-bomb. It’s hitting slowly this time. But I’m still afraid, actually terrified...

Anybody else having situational #paranoia? Unknown noises make me paranoid and the case is worse in cars. I always think it’s breaking...

#### Subtheme 1.2: Medication Experiences

Medication experiences were exchanged between #bipolarclub community members in diverse tweets (53/547, 9.7%). Some members tweeted about the side effects of their medications, whereas others shared experiences, opinions, and advice regarding their effectiveness and adherence:

On Abilify, I gained 15 pounds without changing my diet. These medications lead to metabolic dysfunction. Cut off blaming psychiatric patients!...

@CommunityMember Take your medication and if you have any concerns, talk to your doctor. My doctor recently had to modify my Sertraline...

Not all like to be on medication is understandable, but there is no need to make those who take them, like me, feel as we are lower than. Medication saved my life...

Gosh I forgot to get my meds again...“TAKE YOUR MEDS” #RememberYourMeds...

#### Subtheme 1.3: Health Care System Experiences and Feedback

Members of the #bipolarclub community posted tweets regarding their personal experiences with the health care system (35/547, 6.4%). Some tweets in this discourse subtheme involved their feedback, experiences, and inquiries about the diagnostic and therapeutic process of mental health care, including therapy sessions, diagnostic experiences, interactions with psychiatrists, and aspects of prescriptions and treatment:

4th appointment with a new psychiatrist today. I like her but we only had 15 minutes; thus, not everything was covered. Today, my Invega dosage was increased to help with the hallucinations, but it didn’t address my intrusive thoughts at all...

It was a really good appointment today. The therapist really went to the origin of my struggles with getting up in the morning. She mentioned that my body has a memory of it being traumatic, and it has transformed this memory into a habit. Interesting...

It took 12 years for the correct diagnosis since my first bipolar episode. For bipolar patients this is common... We need more understanding of bipolar disorder...

@CommunityMember Once before, a doctor told me to go for a walk along the coastal path when I was suicidal. The side near us was on a cliff edge! That’s why we need support & peer groups. We need to be there for each other...

... Since I can’t talk to my doctor until tomorrow, I have a question. Blood coagulation and Lamictal, is that normal?

Members of the #bipolarclub community also exchanged experiences and feedback on health insurance and financial assistance programs for individuals with mental health conditions through their tweets:

Because my insurance company won’t pay for my antipsychotic, I’m forced to stop it. Experiences? Any suggestions?...

Have you or a loved one got financial aid from the US government because you have bipolar? Pros & cons? I feel it’s time for me to apply for it and feeling deeply vulnerable...

In addition, #bipolarclub community members used the platform to share helpline contacts of mental health foundations and tag their Twitter accounts. These tweets often included personal experiences with these services and featured hashtags such as #NoShame, aimed at encouraging mental health support seeking and breaking the associated stigma:

I’ve used @samaritans helpline 116 123 #NoShame... Watch my experience with Samaritans countless times...#DepressionIsReal

#### Subtheme 1.4: Treatment Experiences and Recommendations

Another subtheme of discussions within the #bipolarclub community revolved around treatment experiences and recommendations (22/547, 4%). This subtheme consisted of tweets through which the community members openly discussed their personal journeys with different treatments and coping strategies, as well as offered advice based on their experiences:

Using the Chopra app regularly for meditation has been a real game-changer for me over the last couple of years. I feel much less easily triggered and so calmer than before...

Sleep is arguably a major factor in mental health treatments and maintenance. Watch a video made by me about mental health and SLEEP!...

### Theme 2: Daily Life Challenges, Coping Experiences, and Recommendations

#### Overview

The second most frequent discourse theme in the #bipolarclub community, as reflected in its members’ tweets, focused on the everyday challenges of living with mental health conditions, along with coping strategies and recommendations provided through their tweets. This discourse theme involved 17.2% (94/547) of the tweets in total and contained two subthemes: (1) daily life challenges of living with mental health conditions (73/547, 13.3%), which was the most discussed, followed by (2) experiences and recommendations for coping with daily life challenges of living with mental health conditions (21/547, 3.8%), which was addressed to a lesser extent.

Overall, this discourse theme (94/547, 17.2%) generated 5 of the 6 individual-level empowerment processes through the tweets, with the highest proportion related to informational support (92/94, 98%), followed by self-expression support (80/94, 85%), network support (29/94, 31%), emotional support (10/94, 11%), and esteem support (5/94, 5%). In addition, it facilitated the 2 informal carer empowerment processes, with family awareness support leading at 11% (10/94) and friend awareness support at 9% (8/94). Friend awareness support had its highest frequency rate in this theme compared to other themes. This theme also drove the social awareness support process, contributing to this society-level empowerment process in 20% (19/94) of the tweets.

#### Subtheme 2.1: Daily Life Challenges of Living With Mental Health Conditions

Members of the #bipolarclub community tweeted about a range of daily life challenges of living with mental health conditions (73/547, 13.3%). These challenges encompassed various aspects of their lives, including general daily routines, social and family relationships, parenting responsibilities, managing holiday seasons, work commitments, and the pursuit of hobbies or personal interests:

I can’t get out of this “low grade” depression mode. I don’t want to unalive myself. But I can’t be bothered to move, cook, socialize and have no interest in anything. Everything is dull and grey...

When going through a depressive episode, who else avoids friends and then feels so lonely telling yourself that no one cares or understands you?...

It’s difficult when your family don’t want to believe what you go through daily 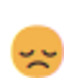...

I wish to offer my daughter a wonderful New Year’s Eve, but getting out of bed is hard. At midnight I’ll be all hypo, I know that...

Many years ago, I talked to a buddy about how terrible for me to go to work when completely anxious and depressed. He asked “What are you going to do about it?”. I was clueless. Every possibility appeared dreadfully unattainable. I continued to suffer until I became suicidal...

On the bright side, I’m writing again after a mentally forced gap...

#### Subtheme 2.2: Experiences and Recommendations for Coping With Daily Life Challenges of Living With Mental Health Conditions

Some members of the #bipolarclub community shared their personal experiences and offered recommendations to address the various aspects of daily life challenges that individuals may encounter while living with mental health conditions (21/547, 3.8%). These insights addressed the same daily life challenges discussed within the community in subtheme 2.1, which included general everyday activities, social and family relationships, parenting duties, navigating holiday periods, work obligations, and engaging in hobbies or personal interests:

An article from a life lesson learned about “How To Live in the Present Moment With Bipolar Disorder”...

Some tips in this post about how to support your children in school as a parent with a mental disorder...

Feeling guilty for not joining the Christmas festivities outside the house. I’m too twitchy and have No energy. Socials are so overwhelming. Telling myself that it is ok. Some people might not realize it, but they won’t hate me for it, I wish...

I haven’t any career so to speak of, but I’ve worked my most life and paid enough National Insurance contributions to earn myself a pension, thank goodness. So even if you have a mental disorder, it is possible...

It’s hard to stay motivated making music and having a mental illness 
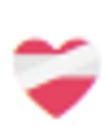
. Sometimes I feel discouraged and don’t want to write or record. But I keep trying to write and record because I know it isn’t true!...

### Theme 3: Socializing and Connecting

#### Overview

Members of the #bipolarclub community did not only tweet about health-related aspects; they also used the OMHC to socialize and engage with one another. This social interaction formed the third most common discourse theme, representing 15.9% (87/547) of the tweets. This theme included 7 subthemes, the highest number of subthemes among all themes of discussions, suggesting that the #bipolarclub community facilitated various social activities and encouraged its members to engage in those activities. The seven subthemes revolved around (1) peer support interactions (27/547, 4.9%), (2) joking about personal mental health and daily life struggles (23/547, 4.2%), (3) Celebrating occasions and special events (21/547, 3.8%), (4) personal artworks (5/547, 0.9%), (5) daily life activities (4/547, 0.7%), (6) praise for the community (4/547, 0.7%), and (7) perspectives on mental health conditions in media and celebrities’ personal experiences (3/547, 0.5%).

In relation to the OMHC population-specific empowerment processes, tweets in this discourse theme (87/547, 15.9%) drove the 6 individual-level empowerment processes. Informational support appeared most frequently (69/87, 79%), followed by network support (65/87, 75%), self-expression support (55/87, 63%), emotional support (41/87, 47%), esteem support (15/87, 17%), and tangible support (11/87, 13%). Network support had its highest prevalence ratio in this discourse theme over other themes. This theme also facilitated family awareness support (3/87, 3%) within the informal carer empowerment process category and social awareness support (6/87, 7%) within the society-level empowerment process category.

#### Subtheme 3.1: Peer Support Interactions

Some tweets by #bipolarclub community members were dedicated to peer support interactions (27/547, 4.9%). These interactions included requests for and offers of support, checking in on one another, and sharing reassuring words to help others feel less alone and more connected, providing comfort in knowing that others encounter similar experiences and challenges [20]:

I can use some support if anybody is available. I’m really struggling...

Has anybody heard from @CommunityMember?...

Thoughts that I’m in a permanent battle with even to make it through the day, it’s unbearable and difficult at times, but always keep in mind that you aren’t alone, every day is a battle, every day is a victory but one day at the time, never give up, I’m here if you need me!...

#### Subtheme 3.2: Joking About Personal Mental Health and Daily Life Struggles

A number of #bipolarclub community members tweeted to share humor about their mental health conditions and everyday challenges (23/547, 4.2%). Tweets within this subtheme featured jokes about personal conditions and symptoms, treatment and medication, social relationships, and even the OMHC itself:

For people with social anxiety, there should be a weather application. “Today will be partly crowdy with a 60% chance of people you know.”...

I went to my appointment yesterday #bipolarclub and I actually had Cymbalta and Seroquel taken off my meds list and now on #Lamictal for bipolar management. There’re more PLUS 
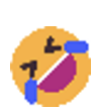
 I actually put myself on for a therapist 
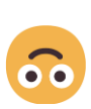

“Are you being productive manic or did you simply get recharged from being on vacation?” 
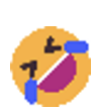

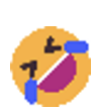
 Oh mom...

#bipolar is trending! Hahaha, well, farewell #bipolarclub. It’s not a club I ever wanted to be a member of anyway.

#### Subtheme 3.3: Celebrating Occasions and Special Events

Diverse tweets from #bipolarclub community members were posted to celebrate different occasions and special events (21/547, 3.8%), including holidays, birthdays, and meaningful moments such as the anniversary of mental health condition diagnosis:

Thank you all for your insightful, comic and interesting tweets. In the new year, I’ll look forward to more interactions. Wishing you all the best for the festive season. Over the next few weeks keep strong #bipolarclub, it’s a testing time, be careful of your triggers and stay safe 
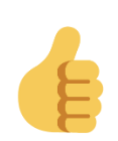

It’s my birthday, I’m so excited...

Happy 1st anniversary to my #bipolardisorder diagnosis 
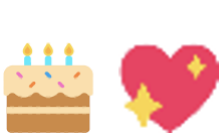
 “Care plan – Diagnosis: bipolar affective disorder... Current medication: Lamotrigine 200mg twice a day... Review date: 22/12/21”...

In addition, other tweets from a peer support foundation, an “organizational member” within the #bipolarclub community [7], were shared to introduce new staff members of the foundation to the OMHC and provide details about online events for those interested in joining:

Join and meet our new Executive Director...

#### Subtheme 3.4: Personal Artworks

Members of the #bipolarclub community also tweeted to share their personal works of art as a way to socialize within the OMHC (5/547, 0.9%). This subtheme involved tweets showcasing members’ drawings and crochet crafts:

I’d never expressed myself through art as I’d like to, but this changed yesterday morning and it feels good. [me, crayon, 2022]...

Headband and beanie I crocheted 
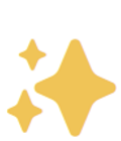
...

#### Subtheme 3.5: Daily Life Activities

Some members discussed their daily life activities within the #bipolarclub community (4/547, 0.7%). In this subtheme of tweets, members mentioned what they were doing at the moment or planned to do:

So #bipolarclub, I’m watching one of my favorite holiday movies, Home Alone 1 & 2, and all I think about is what Kevin basically says “Merry Xmas, you can trust me” to Pigeon Lady...

Taking a break from Twitter to focus more on my project and my #MentalHealth without #bipolarclub. I can’t learn how to control my emotions, so for at least 3 days the max. I 
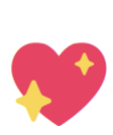
 U! All very much! See you...

#### Subtheme 3.6: Praise for the Community

The positive impact of the #bipolarclub community on its members prompted some to express their appreciation and praise for the OMHC (4/547, 0.7%). Within this discourse subtheme, some tweets encouraged greater participation in the #bipolarclub community, whereas others conveyed gratitude for its role as a valuable resource in helping members cope with both ongoing and immediate mental health challenges:

@CommunityMember Then we’re pretty similar in terms of how long we’ve been diagnosed with bipolar. I hope you’ve had a good year and learned a lot; if not, just stick to the community here on Twitter; we’re nice and tell our experiences 
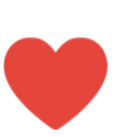
...

Thanks a lot #bipolarclub for making this year more tolerable 
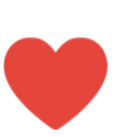

I’m completely a different person today! 
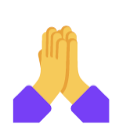
 #bipolarclub 
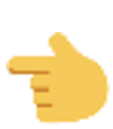
 Okkaayy, there’s a club that could be interesting. 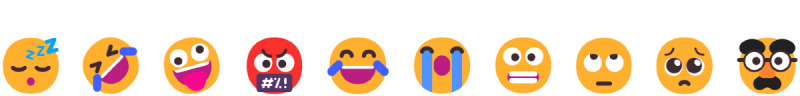

#### Subtheme 3.7: Perspectives on Mental Health Conditions in Media and Celebrities’ Personal Experiences

Some members of the #bipolarclub community tweeted to discuss works related to mental health conditions (3/547, 0.5%), including movies and podcasts, as well as their favorite artists who had mental disorders:

#bipolarclub what do you think of Silver Linings Playbook film?

Who is your preferred artist, musician, author, etc. who has bipolar disorder? Mine is... A quote from her book is here...

### Theme 4: Mental Health Awareness and Stigma Prevention Initiatives

#### Overview

Members of the #bipolarclub community used the platform to raise awareness about mental health conditions and fight their associated stigma through 12.6% (69/547) of the tweets, making this discourse theme the fourth most common one within the online community. Many of the tweets in this theme included relevant trending hashtags on Twitter such as #mentalhealthmatters and #mentalhealthawareness. This theme contained four subthemes of tweets, ranked by prevalence from highest to lowest: (1) personal insights on mental health journeys and stigma (32/547, 5.9%), (2) clarifying misconceptions about mental health conditions and fighting stigma (20/547, 3.7%), (3) groups and events for mental health support and awareness (15/547, 2.7%), and (4) marketing mental health awareness products (2/547, 0.4%).

Overall, tweets in this discourse theme (69/547, 12.6%) enabled all the consumer empowerment processes across the 3 categories of population-specific processes. Notably, this theme was one of only 2 that facilitated all the empowerment processes. Among the individual-level empowerment processes, informational support was the most prevalent in all tweets of this theme (69/69, 100%), with self-expression support (43/69, 62%), network support (36/69, 52%), tangible support (19/69, 28%), emotional support (17/69, 25%), and esteem support (7/69, 10%) next in prevalence. Regarding the informal carer empowerment processes, family awareness support (10/69, 14%) was more common in this discourse theme than friend awareness support (4/69, 6%). Regarding the society-level empowerment process category, social awareness support appeared in 52% (36/69) of the tweets in this theme. Furthermore, 3 consumer empowerment processes—informational support and tangible support from the individual-level category and social awareness support from the society-level category—had their highest frequency ratio in this discourse theme compared to other themes.

#### Subtheme 4.1: Personal Insights on Mental Health Journeys and Stigma

Some of the #bipolarclub community members shared personal insights about their journeys with mental health conditions and stigma (32/547, 5.9%). These tweets included content such as documentaries and articles highlighting personal stories, as well as individual experiences with mental health stigma and suggestions on how to deal with it:

In September 2022, a documentary was shot of me telling my story with bipolar. I never regretted doing this. Watch it...

My life’s hardest times and how a dog rescued it. Watch my TEDX video on mental health and psychiatric service dogs...

The latest published post on my blog. This article is about how psychosis and depression can creep in and how it looks to others from their perspectives...

Hey everybody, my pen name is... One day I’ll have the courage to talk under my real name freely. Unfortunately, my work doesn’t accept mental health struggles, so I use a pen name. I look forward to connecting with those in the #bipolarclub

A recommended post on my blog about “Navigating stigma with a mental illness”...

#### Subtheme 4.2: Clarifying Misconceptions About Mental Health Conditions and Fighting Stigma

Members of the #bipolarclub community also used the platform to clarify misconceptions about mental health conditions based on personal experiences and help destigmatize these conditions (20/547, 3.7%):

@AstrologyAccount Stop this! Making rude little posts comparing bipolar to astrology is despicable and stigmatizing...especially what is written does not describe bipolar disorder at all. Read something...

Another side of #bipolardisorder, we don’t have more than one personality and it isn’t just mood swings. I hope this video reaches somebody who needs to understand this better, love you guys...

#### Subtheme 4.3: Groups and Events for Mental Health Support and Awareness

Some #bipolarclub community members tweeted details about mental health support and awareness groups and events (15/547, 2.7%). Some tweets included announcements regarding the audio support and awareness sessions managed by the #bipolarclub community crew and held on Twitter Spaces through the community’s Twitter account (@BipolarClubDx):

Hey #bipolarclub! Join us today in our Twitter Space #DBTclub!... Topic: Healthy Relationships... Hosts: @CommunityCrewMember1...@CommunityCrewMember2... @CommunityCrewMember3...

In addition, other tweets involved announcements about free online and in-person mental health support and awareness groups and events; some were collaboratively organized by #bipolarclub community members (active advocates within the community) and mental health foundations or entirely hosted by a peer support foundation, whereas others represented initiatives aimed at collaborating on peer support groups:

Making time for Black mental health event and save the date!... Event Date:... 
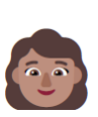
 Meet real Black therapists... A @ThinkTenacity event to freely access Black Therapists... 
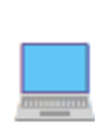
 Group support via Zoom... #BlackWellness

Join us at our 2nd annual holiday music room and have some fun with us 
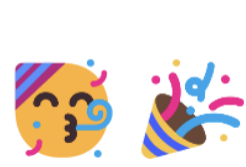
 ... #bipolarmusic

I support proudly @samaritans @CLSamaritans this #BrewMonday...at...332...Road... Don’t forget to remind everyone to reach out for a cuppa and a catch-up with the people you care about... #bluemonday #JanuaryBlues #DepressionIsReal...

Do you have any interest in a support club on Skype or like?...

#### Subtheme 4.4: Marketing Mental Health Awareness Products

In an effort to raise mental health awareness not only in the online environment of the #bipolarclub community but also in the physical world, some members posted tweets advertising their mental health awareness products (2/547, 0.4%), often labeled with phrases such as “bipolar awareness”:

My wristband designs of BIPOLAR :): AWARNESS are made and ready to buy on my Etsy store shortly. This is my first design and I’ll make a bunch more if they’re popular...what do you think?...

### Theme 5: Behavioral Coaching and Motivational Dialogue

#### Overview

The #bipolarclub community was used by its members to exchange behavioral coaching tips and experiences on managing oneself or interactions with others, as well as to share self-motivation thoughts and behaviors for overcoming the challenges of mental health conditions. This discourse theme ranked as the fifth most common topic within the online community, comprising 11.5% (63/547) of the tweets. We defined two subthemes within this discourse theme: (1) behavioral coaching tips and experiences (40/547, 7.3%), which was the most discussed subtheme, and (2) motivational dialogue (23/547, 4.2%).

With regard to the 3 categories of OMHC population-specific empowerment processes, this discourse theme, represented by 11.5% (63/547) of the tweets, was the only one, alongside theme 4 (mental health awareness and stigma prevention initiatives), that enabled all the consumer empowerment processes across these categories. Within this theme of discussion, the 6 individual-level empowerment processes were facilitated, with informational support being the most prominent (61/63, 97%) followed by self-expression support (38/63, 60%), emotional support (36/63, 57%), network support (26/63, 41%), esteem support (16/63, 25%), and tangible support (1/63, 2%). Emotional support and esteem support had their most significant prevalence rate in this discourse theme compared to other themes. In addition, this theme, through its tweets, drove the 2 informal carer empowerment processes—family awareness support in 8% (5/63) of the tweets and friend awareness support in 3% (2/63) of the tweets—as well as the society-level empowerment process, social awareness support, in 11% (7/63) of the tweets.

#### Subtheme 5.1: Behavioral Coaching Tips and Experiences

Some #bipolarclub community members posted tweets that included behavioral coaching tips for managing oneself or relationships with others (40/547, 7.3%), which could help in self-management. Some of these tips were based on personal experiences, whereas others were not:

This morning, I tried out radical acceptance. I chose to accept that I would likely feel anxious instead of wishing I didn’t feel anxious upon waking, and I used affirmations and breathing exercises to get through it...

Good morning, #bipolarclub! 
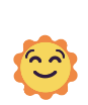
 Learn the 8 ways of how to stop being a chronic people pleaser!...

#### Subtheme 5.2: Motivational Dialogue

The #bipolarclub community served as a platform for some of its members to share their self-motivation thoughts and behaviors related to managing mental health conditions and the challenges they faced in their lives (23/547, 4.2%):

New 2023... Dear Me. 
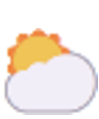
 Keep in mind every morning you’re beautiful. 
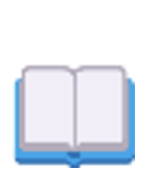
 I will track daily my bipolar moods. 
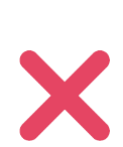
 I won’t put pressure on myself for 2023. 
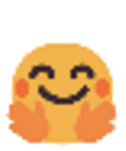
 Never alone struggling with #bipolarLIFE... For SUPPORT @BipolarClubDx @BipolarUK @IntlBipolar...

I can see a bit of light. No matter how small, I can see some light. 
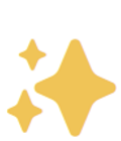
 Getting up and brushing myself off, with the support of friends, family, and the grace of God. One day at a time...

### Theme 6: Personal Feelings, Thoughts, Experiences, and Reflections

#### Overview

The least common discourse theme, shaped by 8.6% (47/547) of the tweets, showed that the #bipolarclub community was used by some of its members as an outlet to express their personal feelings, thoughts, negative experiences, and reflections. This theme encompassed three subthemes of tweets, ordered by frequency from most to least common: (1) personal feelings, thoughts, and reflections (30/547, 5.5%), (2) personal negative experiences and coping struggles (9/547, 1.6%), and (3) hopes and prayers to cope with mental health conditions (8/547, 1.5%).

Overall, this discourse theme (47/547, 8.6%), through its tweets, facilitated 4 of the 6 individual-level empowerment processes, with informational support emerging as the most common (45/47, 96%), followed by self-expression support (44/47, 94%), emotional support (9/47, 19%), and network support (7/47, 15%). It also drove 1 of the 2 informal carer empowerment processes, family awareness support (7/47, 15%), as well as the society-level empowerment process, social awareness support (3/47, 6%). Furthermore, this theme of tweets had the highest ratio of self-expression support within the individual-level empowerment process category and of family awareness support within the informal carer empowerment process category in comparison to the other themes.

#### Subtheme 6.1: Personal Feelings, Thoughts, and Reflections

Some members of the #bipolarclub community tweeted to share their personal feelings, thoughts, and reflections (30/547, 5.5%). Many of these tweets were related to their struggles with mental health challenges, whereas others focused on their perspectives regarding acceptance or support from others:

If I wasn’t Muslim and suicide wasn’t a sin, I won’t probably be here anymore...

How can everything smash in a night, I’m looking at suicide right now...

So, at 4 am I get on the app and scroll through your stories. I do really care. But I couldn’t respond. So that’s me, despondent, discouraged, hurting (a bit), worried, and scared. Ending now this #vent. Good morning. #bipolarclub @BipolarClubDx...

From time to time, I think about people who don’t want to be with me because of me being bipolar, and it leads me to be sad at first, but then I feel like, well, everyone has a short life, they have the right to spend it with a normal person...

So grateful for the people who stand by us. It helps...

#### Subtheme 6.2: Personal Negative Experiences and Coping Struggles

Some members used the #bipolarclub community to discuss personal negative experiences related to family members or their childhood, whereas others shared their struggles in coping with these experiences (9/547, 1.6%):

Why my dad and his wife do still treat me as if I’m incapable of making a decision myself and I’m almost 57!!! Is that down to my #bipolardisorder #bipolarclub?

I’ve just been re-traumatized by my family a week ago... Anyway, New Year, new me...
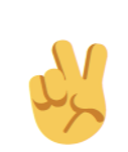
...#toxicfamily...

I keep having recognition more and more about the absolute hell of my childhood and I find it hard to find a way for some heavy feelings of anger...

#### Subtheme 6.3: Hopes and Prayers to Cope With Mental Health Conditions

Some #bipolarclub community members tweeted to express their hopes and prayers for coping with mental health conditions (8/547, 1.5%):

“With great power comes great responsibility”... I wish I had a different life and was never hurt. I wish I could be normal every day as I’m not. Through great suffering, real love and empathy come...

The crash is the worst part of hypomania. And right before Christmas, too. Again! God, how I cope with this feeling...

## Discussion

### Principal Findings

#### Overview

To the best of our knowledge, this is the first study to qualitatively examine how discourse themes within a Twitter-based OMHC, specifically the #bipolarclub community, drive varied population-specific empowerment processes consistent with global empowerment standards (Strategy 1 of the WHO’s IPCHS framework). With this investigation, we provided a deeper understanding of (1) the discourse themes within the community and the extent to which these themes resonate with its members and (2) the population-specific empowerment processes driven by these themes, aligned with the 3 categories of population-specific empowerment processes corresponding to the populations outlined in the WHO’s Strategy 1 [7].

Overall, tweets using the hashtag #bipolarclub encompassed a variety of discourse themes that reflect the inner world of the #bipolarclub community—its members’ experiences, concerns, and discussions. We identified six overarching discourse themes ordered by the extent to which they resonated with #bipolarclub community members from highest to lowest: (1) symptom, medication, treatment, and health care system experiences; (2) daily life challenges, coping experiences, and recommendations; (3) socializing and connecting; (4) mental health awareness and stigma prevention initiatives; (5) behavioral coaching and motivational dialogue; and (6) personal feelings, thoughts, experiences, and reflections. In addition, we revealed that #bipolarclub community members, through mental health awareness–related discussions (discourse theme 4), extended their efforts beyond the online environment into real-world settings. This finding demonstrates that the OMHC awareness discourse can not only engage online members but also reach a wider audience of health care consumers from various populations in the physical world.

Our results also showed that all 6 discourse themes generated empowerment processes to varying degrees across the 3 categories of population-specific empowerment processes, with only themes 4 and 5 driving all the empowerment processes within these categories. This finding underscores the multifaceted roles of the #bipolarclub community’s diverse discourse themes in generating (1) individual-level empowerment processes for those with mental health conditions, including underserved and marginalized individuals; (2) informal carer empowerment processes for their family members and friends; and (3) society-level empowerment processes for the broader public. It also suggests that the different themes discussed—covering health-related, personal, and social aspects—within a single OMHC have the potential to empower various populations, particularly those identified in the WHO’s Strategy 1, thereby advancing global empowerment objectives. The variation in how discourse themes drive diverse population-specific empowerment processes highlights the multiple roles that these themes play in addressing the specific support needs of various populations. Furthermore, our finding emphasizes the significant role of themes 4 and 5 in comprehensively fostering empowerment across various populations of health care consumers.

In addition, our analysis unveiled that different discourse themes strongly drove the empowerment processes across the 3 categories of population-specific empowerment processes within the #bipolarclub community. For instance, informational support, the most prevalent individual-level process within the community’s discussions, and social awareness support, the main society-level process, were strongly driven by theme 4, whereas the 2 informal carer processes, family awareness support and friend awareness support, were primarily driven by themes 6 and 2, respectively. These findings underscore the influential role of discourse themes in shaping the type of empowerment generated in OMHCs.

#### Discourse Themes in OMHC Discussions and Population-Specific Empowerment Processes Driven by OMHC Discourse Themes

The discourse theme *symptom, medication, treatment, and health care system experiences* emerged as the most frequently discussed theme within the #bipolarclub community, which underscores its significance in the community’s discussions. In addition, experiential informational support—an empowerment subprocess of informational support within the individual-level category of empowerment processes—was highly prominent in this theme relative to the other themes. These findings could be attributed to gaps in traditional mental health services and resources [3,17,41], as well as to a preference of #bipolarclub community members to share and gain insights from one another’s personal experiences navigating mental health challenges. This preference aligns with one of the community’s key objectives, “learning from one another” [59], and echoes the findings of a recent study [6]. This study, which examined TikTok videos related to depression and anxiety, found that, while symptoms were the most frequently discussed topic in both personal experience and health care expert videos, the engagement was higher with personal experience videos compared to those by experts [6]. In line with this study [6] and another study on Reddit OMHCs [14], our results confirm the prevalence of discussions related to, for example, symptom, medication, treatment, and health care system experiences and the frequent exchange of personal experience–based information in these discussions. However, our study makes a notable contribution by underscoring the pivotal role of the identified discourse theme in driving experiential informational support, which is a key element in fostering individual-level empowerment within this context.

The second most prevalent discourse theme in the #bipolarclub community was related to *daily life challenges, coping experiences, and recommendations*, encompassing everyday challenges of living with mental health conditions as well as personal experiences and recommendations to cope with these challenges, such as general daily activities, social and family relationships, parenting duties, managing holiday seasons, work obligations, and pursuing hobbies or personal interests. Our results revealed that, while coping strategies were actively discussed, discussions regarding the challenges themselves were more prominent. This finding suggests a potential gap in available coping strategies within the #bipolarclub community for those facing these challenges, which highlights a need for mental health experts to provide more guidance within the OMHC. This insight aligns with those of a recent study that interviewed OMHC moderators, revealing a preference and need for expert involvement, particularly to offer personalized support and identify users needing immediate attention [9]. It also supports previous research that has reported such daily life challenges as common discussion topics in OMHCs [37,38]. In addition, our analysis showed that this discourse theme within the #bipolarclub community drove family awareness support and friend awareness support (the 2 empowerment processes in the informal carer category), with friend awareness support being especially prominent in this theme compared to other themes. This finding reinforces those of previous research that highlights the significant role of close relationships in addressing the daily challenges faced by individuals with mental health conditions, extending support beyond family to include friends. For example, a recent study on bipolar disorder communities on Reddit found that individuals with mental health conditions expressed the need for support from their family and friends [14]. Our finding further emphasizes the valuable role that OMHCs play in driving empowerment processes for family and friends. By drawing on insights from individuals with lived experiences, these discussions help deepen family and friends’ understanding of the challenges faced, thus equipping them to become more actively engaged in mental health support practices.

In addition, our analysis demonstrated that #bipolarclub community members were highly engaged in a wide range of discussions under the theme of *socializing and connecting*, which included 7 subthemes—the most among the 6 main themes identified. This finding correlates with our observation that network support, an individual-level empowerment process, was particularly prevalent in this theme. While previous studies have indicated that OMHCs are often used for socializing and connecting, which in turn fosters network support [20,22,54,69], our findings demonstrate that the #bipolarclub community not only embraces these functions but also cultivates a rich and comprehensive socializing environment through multiple forms of social interaction–focused discussions. This insight highlights the potential of OMHCs to offer an enriched social experience.

Our findings also revealed that *mental health awareness and stigma prevention initiatives* commonly appeared as a key discourse theme within the #bipolarclub community. Similar discussions to those identified in this theme have also been highlighted in previous studies, such as sharing personal stories about mental health, disseminating personal experiences of stigma, and providing explanations for mental health conditions [4,11]. However, our study showed that the #bipolarclub community’s awareness and stigma prevention efforts extend beyond these previous findings, especially in offering *groups and events for mental health support and awareness* and *marketing mental health awareness products*. The community organized free online and in-person support and awareness groups and events, as well as hosted audio discussions via Twitter Spaces. Some of these initiatives were conducted in collaboration with mental health foundations. Also, the community promoted mental health awareness products (eg, wristbands) labeled with “bipolar awareness.” These efforts exemplify how the #bipolarclub community extends its awareness initiatives’ discussions beyond the confines of its online environment, bridging the digital and physical worlds to engage broader audiences. To define this phenomenon, we introduce the concept of *OMHC mass awareness discussions*, where awareness discussions originating in OMHCs expand their reach to involve wider audiences from diverse populations of health care consumers in offline settings. This approach could actively include individuals who may face barriers to accessing OMHCs, such as limited internet access and digital literacy challenges, thereby broadening the impact of these discussions.

In addition, our findings showed that theme 5 (*behavioral coaching and motivational dialogue*) and theme 6 (*personal feelings, thoughts, experiences, and reflections*) were identified as the 2 least pervasive discourse themes within the #bipolarclub community. Theme 5 focused on behavioral coaching tips and experiences for managing personal behaviors or relationships with others, as well as self-motivation thoughts and behaviors to overcome mental health challenges. Theme 6 centered on personal expressions of feelings, thoughts, and reflections related to mental health struggles; discussions about negative experiences with family members or childhood; and personal expressions of hopes and prayers to cope with mental health conditions. Both themes commonly featured elements of self-motivation, self-disclosure, self-expression, venting, and prayer. However, self-expression support, an individual-level empowerment process, was particularly prominent in theme 6 more so than in theme 5 or any other themes, aligning with its introspective discussions. In addition, family awareness support, an informal carer empowerment process, was more prevalent in theme 6 compared to all other themes, likely due to its focus on family-related experiences. Despite being centered on negative family interactions, the discussions in theme 6 offer valuable insights for families, helping them better understand the lived experiences of individuals with mental health conditions and enabling their families to provide more effective support. A Twitter-based OMHC can further foster this process by offering its members greater anonymity, encouraging more openness without fear of judgment—unlike platforms such as Facebook, where connections often involve real-life acquaintances, making it more difficult to openly share personal experiences related to family members [37,54,70].

While previous research has acknowledged the presence of self-talk, interpersonal conversations, and prayers in OMHC discussions on social media and their significant role in empowering health care consumers [3,4,11,20,37,54], limited attention has been paid to identifying the specific types of discourse communication that drive this empowerment. Our findings, consistent with those of previous research, revealed that the #bipolarclub community encompassed different types of discussions, including self-discussions (eg, self-motivation, self-disclosure, and venting), interpersonal discussions (with other community members), and spiritual discussions (eg, praying). This result suggests that the OMHC facilitates 3 distinct forms of empowerment discourse communication for its members. Building on this finding and previous literature, we propose a new framework—the *OMHC empowerment discourse communication classification*—which categorizes the empowerment discourse communication within OMHC discussions into three key types: (1) *intrapersonal discourse*, (2) *interpersonal discourse*, and (3) *spiritual discourse*. Table 3 provides detailed descriptions of these discourse types accompanied by sample paraphrased tweets from discussions within the #bipolarclub community.

**Table 3:** A classification of the 3 types of empowerment discourse communication within OMHC discussions. OMHC: online mental health community.

Type of empowerment discourse communication	Description	Example
Intrapersonal discourse	Intrapersonal discourse in OMHCs refers to internal communication or self-directed dialogue, involving self-motivation, self-disclosure, self-expression, venting, and personal reflection, enabling individuals to process their emotions, mental health–related challenges, and experiences through introspection, often serving as a means of emotional release or gaining clarity.	I can’t get out of this “low grade” depression mode. I don’t want to unalive myself. But I can’t be bothered to move, cook, socialize and have no interest in anything. Everything is dull and grey...
Interpersonal discourse	Interpersonal discourse in OMHCs refers to communication directed toward OMHC members, encompassing various forms such as sharing information or personal experiences, seeking or offering advice, providing emotional support, building connections, and advocating for mental health, all of which help foster a sense of connection and collective support among OMHC members.	@CommunityMember Take your medication and if you have any concerns, talk to your doctor. My doctor recently had to modify my Sertraline...
Spiritual discourse	Spiritual discourse in OMHCs refers to religious or spiritual communication, including prayers, expressions of faith, and spiritual reflections directed toward God, used as a coping mechanism or means of seeking guidance and strength in the context of mental health challenges.	The crash is the worst part of hypomania. And right before Christmas, too. Again! God, how I cope with this feeling...

### Theoretical and Practical Implications

This study contributes to a deeper understanding of consumer empowerment in OMHCs. It provides valuable insights into how discourse themes within these communities drive varied population-specific empowerment processes in line with global empowerment standards, particularly Strategy 1 of the WHO’s IPCHS framework. The key insights from our study are as follows: First, we identified the discourse themes within an OMHC and defined the extent to which these themes resonate with its members, revealing the internal dynamics of such communities. Second, we determined the varied population-specific empowerment processes driven by OMHC discourse themes, demonstrating that all themes within a single OMHC generate empowerment processes to different extents across the 3 categories of population-specific empowerment processes corresponding to Strategy 1 of the WHO’s IPCHS framework (individual-level category, informal carer category, and society-level category). Moreover, we clarified that mental health awareness and behavior–related themes are especially crucial for driving all the empowerment processes within these 3 categories, thereby promoting consumer empowerment comprehensively across various populations. Third, in addition, we proved that the diverse discourse themes within a single OMHC drive empowerment processes for various populations, including individuals with mental health conditions (and those from underserved and marginalized populations), family members and friends (informal carers), and the broader public (society), shedding light on OMHCs’ potential in creating a comprehensive empowering environment consistent with the WHO’s empowerment objectives.

Furthermore, this study introduces new concepts and frameworks. First, we identified *OMHC population-specific empowerment processes* as a new notion, clarifying that OMHCs involve consumer empowerment processes for diverse populations addressing each population’s specific empowerment needs. Second, we defined *OMHC mass awareness discussions* as a new concept, demonstrating that mental health awareness–related discussions within OMHCs extend beyond the online platform to engage wider audiences in offline settings. Third, we proposed the *OMHC empowerment discourse communication classification*, highlighting the 3 types of empowerment discourse communication within OMHC discussions: *intrapersonal*, *interpersonal*, and *spiritual discourses*. Finally, we presented a conceptual framework that links the 6 identified OMHC discourse themes to the 3 categories of OMHC population-specific empowerment processes (individual-level, informal carer, and society-level processes) corresponding to the populations outlined in Strategy 1 of the WHO’s IPCHS framework.

The concepts and frameworks introduced in this study establish a novel foundation for future research on consumer empowerment within OMHCs. For example, researchers can build on our proposed *OMHC mass awareness discussions* concept to further explore the dynamics of mental health awareness within these communities. Also, the *OMHC empowerment discourse communication classification* can serve as a framework to analyze OMHC discussions, focusing on the psychological (intrapersonal), social (interpersonal), and religious (spiritual) types of discourse communication within OMHCs. Such analyses have the potential to advance research on mental health empowerment in OMHCs.

In addition, the findings of this study offer significant benefits for various health care stakeholders, including health care organizations, professionals, and OMHC moderators. For example, governmental and nongovernmental health care organizations (eg, the WHO) can benefit from understanding the capability of diverse discourse themes within OMHCs to drive consumer empowerment processes for various populations aligning with global empowerment standards (Strategy 1 of the WHO’s IPCHS framework). Consequently, they can formulate guidelines and strategies that effectively integrate OMHCs into mental health care systems, positioning these communities as a vital element in more holistic and inclusive care models to promote consumer empowerment in mental health. Additionally, health care organizations, professionals, and OMHC moderators can leverage our findings to enhance their engagement strategies within these communities by generating more targeted and impactful mental health discussions that address the specific empowerment needs of diverse populations. These discussions can focus on specific discourse themes that are either tailored to strongly drive particular empowerment processes for certain populations or designed to comprehensively drive broad empowerment processes for multiple populations. For example, the theme *personal feelings, thoughts, experiences, and reflections* can be used to boost the empowerment process of self-expression support for individuals with mental health conditions, including those from underserved and marginalized populations, and the empowerment process of family awareness support for their families. Conversely, the theme *mental health awareness and stigma prevention initiatives* can be used to comprehensively drive various empowerment processes for diverse populations, benefiting individuals with mental health conditions, underserved and marginalized individuals, family members, friends, and the broader public.

### Limitations and Future Research

While this study provides valuable insights into the dynamics of consumer empowerment within OMHCs, it is essential to acknowledge certain limitations. Our data sample did not include all discussions within the #bipolarclub community during the 4-week study period; it covered only tweets containing the hashtag #bipolarclub, including the hashtag #DBTclub. Consequently, we were unable to capture reply tweets because they often do not include hashtags. Although this limitation means that some perspectives may have been overlooked, concentrating on tweets with the specified hashtag allowed for a more in-depth analysis of the discussions intended to inform the community about relevant content. In addition, we did not consider the online support and awareness groups and events referenced in the analyzed tweets, particularly the public audio conversations hosted on Twitter Spaces by the community’s account. While this omission may have restricted our understanding of discourse themes and population-specific empowerment processes within the #bipolarclub community, it also allowed us to provide in-depth insights particularly related to posts’ communications. Future studies could build on our findings by investigating these audio conversations to better understand the interplay between discourse themes and population-specific empowerment processes within these interactions. Finally, it is important to note that the #bipolarclub community on Twitter may not fully represent all OMHCs or those on other social media platforms. However, this study serves as a pioneering effort in its research area and can guide future investigations into other OMHCs across different platforms to enhance the validity and generalizability of our findings.

### Conclusions

By investigating how discourse themes drive varied population-specific empowerment processes within the #bipolarclub community, we highlight the multifaceted roles of Twitter-based OMHC discourse themes in generating the 3 categories of population-specific empowerment processes corresponding to the various populations outlined in Strategy 1 of the WHO’s IPCHS framework. These populations include individuals with mental health conditions as well as those from underserved and marginalized populations, their informal carers (family members and friends), and the broader public (society). Our analysis demonstrates that all discourse themes within a single Twitter-based OMHC—covering health-related, personal, and social aspects—drive empowerment processes to varying degrees across the 3 categories of population-specific empowerment processes: individual-level, informal carer, and society-level processes. This underscores the capability of the diverse discourse themes in such OMHCs to foster a holistic empowering environment aligned with global standards, highlighting the potential benefits of leveraging Twitter-based OMHCs to support the WHO’s empowerment agenda and advance mental health empowerment worldwide. Given the widespread use of OMHCs and Twitter, along with the vital role of their discourse themes in driving empowerment processes for various populations of health care consumers, these online platforms hold the promise not only to serve as a tool for delivering mental health services but also to create a comprehensive supportive ecosystem for mental well-being. This study offers valuable insights for health care stakeholders, such as health care organizations, professionals, and OMHC moderators, aiding them in developing more tailored, targeted, and effective consumer-empowering strategies within OMHCs for diverse populations.

## Appendix

Multimedia Appendix 1 Sample paraphrased #bipolarclub tweets within the defined discourse themes and subthemes mapped to the three categories of OMHC population-specific empowerment processes: (1) individual-level empowerment processes (for individuals with mental health conditions, including those from underserved and marginalized populations); (2) informal carer empowerment processes (for family members and friends); and (3) society-level empowerment processes (for the broader public). OMHC: online mental health community.

## Abbreviations

API: application programming interface
DBT: dialectical behavior therapy
GIF: Graphics Interchange Format
IPCHS: Integrated People-Centred Health Services
OMHC: online mental health community
RQ: research question
WHO: World Health Organization

## Data Availability

The dataset used in this study is publicly available on Twitter and can be gathered as outlined in the Methods section. However, it cannot be shared directly by the corresponding author due to the sensitive nature of the tweets, which could reveal personal information and compromise the privacy of #bipolarclub community members.
