# Supplementary material for: Understanding How Discourse Themes in an Online Mental Health Community on Twitter/X Drive Varied Population-Specific Empowerment Processes in Alignment With Global Standards: A Qualitative Analysis of #BipolarClub
Source: J Med Internet Res. 2025 Aug 29;27:e74912. doi: 10.2196/74912 (PMC12432477; doi:10.2196/74912)
Supplement: Multimedia Appendix 1 [file jmir_v27i1e74912_app1.pdf]

Multimedia Appendix 1

Sample paraphrased #bipolarclub tweets within the defined discourse themes and subthemes mapped to the three categories of OMHC population-specific empowerment processes: (1) individual-level empowerment processes (for individuals with mental health conditions, including those from underserved and marginalized populations); (2) informal carer empowerment processes (for family members and friends); and (3) society-level empowerment processes (for the broader public). OMHC: online mental health community.

Theme and subtheme	Sample paraphrased tweet	OMHC population-specific empowerment processes		
		Individual-level empowerment processes	Informal carer empowerment processes	Society-level empowerment processes
**Symptom, medication, treatment, and health care system experiences**				
Symptom experiences	Feeling the depression returning. I think I should feel thankful this time that it didn't drop as usual like A-bomb. It's hitting slowly this time. But I'm still afraid, actually terrified. #bipolarclub #depression #mentalillness	• Informational support<br>○ Experiential informational support<br>• Self-expression support		
	Being depressed again. I'm so sick of this condition that I'm on THE VERGE of seeking electroconvulsive therapy or even deep brain stimulation (brain surgery). I'm so tired I want to try any and all available treatments to put an end to this disease! #Bipolar #bipolarclub #mentalhealth	• Informational support<br>○ Experiential informational support<br>• Self-expression support		
	Anybody else having situational #paranoia? Unknown noises make me paranoid and the case is	• Informational support<br>○ Experiential informational support		

	worse in cars. I always think it's breaking. #bipolarclub #bipolar #adhd #ptsd	• Self-expression support<br>• Network support		
Medication experiences	On Abilify, I gained 15 pounds without changing my diet. These medications lead to metabolic dysfunction. Cut off blaming psychiatric patients! #MedTwitter #psychtwitter #bipolarclub	• Informational support<br>○ Experiential informational support<br>• Self-expression support<br>• Esteem support		• Social awareness support<br>○ Perceptive awareness support
	I'm not sure if I could be able to stay on the Trintellix. I don't know what I should do. I feel so sedated. I'm so sick of this. I hate this. #bipolarclub	• Informational support<br>○ Experiential informational support<br>• Self-expression support		
	Did anyone of you #bipolarclub experience an episode despite taking medication? I recently did and it's strange and annoying.	• Informational support<br>○ Experiential informational support<br>• Self-expression support<br>• Network support		
	@CommunityMember Take your medication and if you have any concerns, talk to your doctor. My doctor recently had to modify my Sertraline. #bipolarclub	• Informational support<br>○ Experiential informational support<br>○ Objective informational support<br>• Emotional support		
	Not all like to be on medication is understandable, but there is no need to make those who take them, like me, feel as we are lower than. Medication saved my life #bipolar #bipolarclub	• Informational support<br>○ Experiential informational support<br>• Self-expression support		

	I can’t stop my meds because I will die. I’m a slave to them. What a Dilemma. #bipolarclub #Bipolar	• Informational support<br>○ Experiential informational support<br>• Self-expression support		
	Gosh I forgot to get my meds again…“TAKE YOUR MEDS” #RememberYourMeds #bipolarclub	• Informational support<br>○ Experiential informational support<br>○ Objective informational support<br>• Self-expression support		
Health care system experiences and feedback	4th appointment with a new psychiatrist today. I like her but we only had 15 minutes; thus, not everything was covered. Today, my Invega dosage was increased to help with the hallucinations, but it didn’t address my intrusive thoughts at all. #bipolarclub #sicknotweak #OCD #PTSD	• Informational support<br>○ Experiential informational support<br>• Self-expression support<br>• Esteem support		
	I have never been more happy to have a therapy session this week. I’m just excited to speak to somebody about my life changes recently ❤️ #bipolarclub	• Informational support<br>○ Experiential informational support<br>• Self-expression support<br>• Emotional support		
	It was a really good appointment today. The therapist really went to the origin of my struggles with getting up in the morning. She mentioned that my body has a memory of it being traumatic, and it has transformed this memory into a habit. Interesting. #bipolarclub	• Informational support<br>○ Experiential informational support<br>○ Objective informational support<br>• Self-expression support		

	It took 12 years for the correct diagnosis since my first bipolar episode. For bipolar patients this is common... We need more understanding of bipolar disorder... #bipolar #bipolarclub #MentalHealthMonday	• Informational support<br>○ Experiential informational support<br>• Self-expression support		
	Yesterday, during my psychiatric visit of the end of the year, I asked about autism & ADHD and how they figure in my bipolar 1 mind. She said ADHD is diagnosed in childhood and more than likely turns into bipolar in adulthood. Is that sound right? #adhd #bipolar #bipolarclub	• Informational support<br>○ Experiential informational support<br>○ Objective informational support<br>• Network support<br><br>➢ *Support individuals from underserved and marginalized populations (children)*		
	My appointment with the new doctor was really well. She listened & boundaries were set on both sides. I'm fine with our plan for the next month. I really feel ok with this. #bipolarclub	• Informational support<br>○ Experiential informational support<br>• Self-expression support		
	I'm finally going to take the advice of many and go to a new psychiatrist after spending 7 years going to the same one. I'm really unsure. Any advice? #bipolarclub #bipolar #bipolarcommunity	• Informational support<br>○ Experiential informational support<br>• Self-expression support<br>• Network support		

	@CommunityMember Once before, a doctor told me to go for a walk along the coastal path when I was suicidal. The side near us was on a cliff edge! That’s why we need support & peer groups. We need to be there for each other #bipolarclub	• Informational support<br>○ Experiential informational support<br>• Self-expression support<br>• Network support<br>• Emotional support<br>• Esteem support		
	#bipolardisorder #bipolar #bipolarclub Since I can’t talk to my doctor until tomorrow, I have a question. Blood coagulation and Lamictal, is that normal?	• Informational support<br>○ Experiential informational support<br>• Self-expression support<br>• Network support		
	I just found out that my insurance company won’t cover a medication that has made my life possible. #bipolar #bipolarclub #ANTHEM	• Informational support<br>○ Experiential informational support<br>• Self-expression support		
	Because my insurance company won’t pay for my antipsychotic, I’m forced to stop it. Experiences? Any suggestions? #bipolar #bipolarclub #MentalHealthAwareness	• Informational support<br>○ Experiential informational support<br>• Self-expression support<br>• Network support		
	Have you or a loved one got financial aid from the US government because you have bipolar? Pros & cons? I feel it’s time for me to apply for it and feeling deeply vulnerable #Bipolar #bipolarclub #MentalHealthMatters #MentalHealth #MentalHealthAwareness	• Informational support<br>○ Experiential informational support<br>○ Objective informational support<br>• Self-expression support		

		• Network support		
	I've used @samaritans helpline 116 123 #NoShame… Watch my experience with Samaritans countless times #MentalHealthMatters #mentalhealthsupport #bipolarclub #DepressionIsReal	• Informational support<br>○ Experiential informational support<br>○ Objective informational support<br>• Self-expression support<br>• Network support<br>• Emotional support<br>• Esteem support<br>• Tangible support		• Social awareness support<br>○ Perceptive awareness support<br>○ Destigmatization support
Treatment experiences and recommendations	Using the Chopra app regularly for meditation has been a real game-changer for me over the last couple of years. I feel much less easily triggered and so calmer than before. #bipolardisorder #bipolarclub	• Informational support<br>○ Experiential informational support<br>• Self-expression support		
	Sleep is arguably a major factor in mental health treatments and maintenance. Watch a video made by me about mental health and SLEEP!... #bipolarclub #sleep #MedTwitter	• Informational support<br>○ Experiential informational support<br>○ Objective informational support		
**Daily life challenges, coping experiences, and recommendations**				
Daily life challenges of living with mental health conditions	I can't get out of this "low grade" depression mode. I don't want to unalive myself. But I can't be bothered to move, cook, socialize and have no	• Informational support<br>○ Experiential informational support		• Social awareness support<br>○ Perceptive awareness support

	interest in anything. Everything is dull and grey. #bipolarclub #bipolardisorder #bp2 #depression	• Self-expression support		
	When going through a depressive episode, who else avoids friends and then feels so lonely telling yourself that no one cares or understands you? #bipolardisorder #bipolarclub	• Informational support<br>○ Experiential informational support<br>• Self-expression support<br>• Network support	• Friend awareness support	
	It’s difficult when your family don’t want to believe what you go through daily 😞 #bipolar #bipolarclub	• Informational support<br>○ Experiential informational support<br>• Self-expression support	• Family awareness support	
	I wish to offer my daughter a wonderful New Year’s Eve, but getting out of bed is hard. At midnight I’ll be all hypo, I know that… #bipolar #bipolarclub	• Informational support<br>○ Experiential informational support<br>• Self-expression support	• Family awareness support	
	My brain was totally messed up on Christmas Day. So glad it’s over… #bipolarclub #eupd	• Informational support<br>○ Experiential informational support<br>• Self-expression support		
	Many years ago, I talked to a buddy about how terrible for me to go to work when completely anxious and depressed. He asked “What are you going to do about it?”. I was clueless. Every possibility appeared dreadfully unattainable. I continued to suffer until I became suicidal. #bipolarclub	• Informational support<br>○ Experiential informational support<br>• Self-expression support	• Friend awareness support	• Social awareness support<br>○ Perceptive awareness support

	On the bright side, I'm writing again after a mentally forced gap... #writerslife #bipolarclub	• Informational support<br>○ Experiential informational support<br>• Self-expression support<br>• Emotional support		
Experiences and recommendations for coping with daily life challenges of living with mental health conditions	An article from a life lesson learned about "How To Live in the Present Moment With Bipolar Disorder" #medium #mediumwriters #bipolar #mentalhealth #bipolarclub #WritingCommunity	• Informational support<br>○ Experiential informational support<br>○ Objective informational support		
	Some tips in this post about how to support your children in school as a parent with a mental disorder #bipolarclub	• Informational support<br>○ Objective informational support		
	Feeling guilty for not joining the Christmas festivities outside the house. I'm too twitchy and have No energy. Socials are so overwhelming. Telling myself that it is ok. Some people might not realize it, but they won't hate me for it, I wish. #bipolarclub #bipolardisorder	• Informational support<br>○ Experiential informational support<br>• Self-expression support<br>• Emotional support<br>• Esteem support		• Social awareness support<br>○ Perceptive awareness support
	I haven't any career so to speak of, but I've worked my most life and paid enough National Insurance contributions to earn myself a pension, thank goodness. So even if you have a mental disorder, it is possible #bipolardisorder #bipolarclub	• Informational support<br>○ Experiential informational support<br>• Self-expression support<br>• Emotional support<br>• Esteem support		• Social awareness support<br>○ Perceptive awareness support
	It's hard to stay motivated making music and having a mental illness ❤️‍🩹 . Sometimes I feel discouraged	• Informational support		

	and don't want to write or record. But I keep trying to write and record because I know it isn't true! #MentalHealth #bipolarclub #bipolar	○ Experiential informational support<br>• Self-expression support<br>• Emotional support		
**Socializing and connecting**				
Peer support interactions	I can use some support if anybody is available. I'm really struggling. #bipolarclub	• Informational support<br>○ Experiential informational support<br>• Self-expression support<br>• Network support		
	Anybody from #bipolarclub want to be mutuals? My DMs are available 😊 I adore hearing from everyone of you!	• Network support<br>• Emotional support<br>• Tangible support		
	How are my #bipolar buddies doing? #bipolarclub	• Network support<br>• Emotional support		
	Has anybody heard from @CommunityMember? #bipolarclub	• Network support<br>• Emotional support		
	Thoughts that I'm in a permanent battle with even to make it through the day, it's unbearable and difficult at times, but always keep in mind that you aren't alone, every day is a battle, every day is a victory but one day at the time, never give up, I'm here if you need me! #bipolardisorder #bipolarclub	• Informational support<br>○ Experiential informational support<br>○ Objective informational support<br>• Self-expression support<br>• Network support<br>• Emotional support		

		• Tangible support		
Joking about personal mental health and daily life struggles	For people with social anxiety, there should be a weather application. “Today will be partly crowdy with a 60% chance of people you know.” #bipolarclub #jokes	• Informational support<br>○ Experiential informational support<br>• Self-expression support		• Social awareness support<br>○ Perceptive awareness support
	I went to my appointment yesterday #bipolarclub and I actually had Cymbalta and Seroquel taken off my meds list and now on #Lamictal for bipolar management. There’re more PLUS 🤣 I actually put myself on for a therapist 🙃	• Informational support<br>○ Experiential informational support<br>• Self-expression support		
	“Are you being productive manic or did you simply get recharged from being on vacation?” 🤣 🤣 Oh mom... #bipolarclub	• Informational support<br>○ Experiential informational support<br>• Self-expression support	• Family awareness support	
	#bipolar is trending! Hahaha, well, farewell #bipolarclub. It’s not a club I ever wanted to be a member of anyway.	• Informational support<br>○ Objective informational support<br>• Self-expression support		
Celebrating occasions and special events	Thank you all for your insightful, comic and interesting tweets. In the new year, I’ll look forward to more interactions. Wishing you all the best for the festive season. Over the next few weeks keep strong #bipolarclub, it’s a testing time, be careful of your triggers and stay safe 👍	• Informational support<br>○ Objective informational support<br>• Self-expression support<br>• Network support<br>• Emotional support<br>• Esteem support		
	It’s my birthday, I’m so excited #bipolarclub	• Self-expression support		

		• Emotional support		
	Happy 1st anniversary to my #bipolardisorder diagnosis 🎂 💖 “Care plan – Diagnosis: bipolar affective disorder… Current medication: Lamotrigine 200mg twice a day… Review date: 22/12/21” #bipolar #bipolarclub #mentalhealth #mentalillness	• Informational support<br>○ Experiential informational support<br>• Self-expression support<br>• Emotional support		
	Join and meet our new Executive Director… #bipolar #bipolarclub	• Informational support<br>○ Objective informational support<br>• Network support		
Personal artworks	I’d never expressed myself through art as I’d like to, but this changed yesterday morning and it feels good. [me, crayon, 2022]… #bipolarclub	• Informational support<br>○ Experiential informational support<br>• Self-expression support		
	Headband and beanie I crocheted ✨… #crochet #MentalHealthAwareness #bipolarclub #anxietydisorders #creativity #MentalHealthMatters #beyou	• Informational support<br>○ Experiential informational support<br>○ Objective informational support<br>• Self-expression support<br>• Emotional support		
Daily life activities	So #bipolarclub, I’m watching one of my favorite holiday movies, Home Alone 1 & 2, and all I think about is what Kevin basically says “Merry Xmas, you can trust me” to Pigeon Lady…	• Self-expression support<br>• Network support		

	Taking a break from Twitter to focus more on my project and my #MentalHealth without #bipolarclub. I can’t learn how to control my emotions, so for at least 3 days the max. I 💖 U! All very much! See you...	• Informational support<br>○ Experiential informational support<br>• Self-expression support<br>• Network support<br>• Emotional support<br>• Esteem support		
Praise for the community	@CommunityMember Then we’re pretty similar in terms of how long we’ve been diagnosed with bipolar. I hope you’ve had a good year and learned a lot; if not, just stick to the community here on Twitter; we’re nice and tell our experiences ❤️ #bipolar #bipolarclub	• Informational support<br>○ Experiential informational support<br>○ Objective informational support<br>• Network support<br>• Emotional support<br>• Esteem support		
	Thanks a lot #bipolarclub for making this year more tolerable ❤️	• Self-expression support<br>• Network support<br>• Emotional support<br>• Esteem support		
	I’m completely a different person today! 🙏 #bipolarclub 👈 Okkaayy, there’s a club that could be interesting. 😴 🤣 🤪 🤬 😂 😭 😬 🙄 🥺 🥸	• Informational support<br>○ Experiential informational support<br>• Self-expression support<br>• Network support<br>• Esteem support		
Perspectives on mental health	#bipolarclub what do you think of Silver Linings Playbook film?	• Network support		

conditions in media and celebrities' personal experiences	Who is your preferred artist, musician, author, etc. who has bipolar disorder? Mine is... A quote from her book is here... #bipolarclub	• Informational support<br>○ Objective informational support<br>• Self-expression support<br>• Network support		
**Mental health awareness and stigma prevention initiatives**				
Personal insights on mental health journeys and stigma	In September 2022, a documentary was shot of me telling my story with bipolar. I never regretted doing this. Watch it... #bipolar #bipolarclub #bipolardisorder #MentalHealthAwareness #mentalillness #suicide	• Informational support<br>○ Experiential informational support<br>○ Objective informational support<br>• Self-expression support<br>• Emotional support		• Social awareness support<br>○ Perceptive awareness support
	My life's hardest times and how a dog rescued it. Watch my TEDX video on mental health and psychiatric service dogs... #mentalhealthintheworkplace #mentalhealthmatters #mentalhealthawareness #bipolarclub	• Informational support<br>○ Experiential informational support<br>○ Objective informational support<br>• Self-expression support		• Social awareness support<br>○ Perceptive awareness support
	The latest published post on my blog. This article is about how psychosis and depression can creep in and how it looks to others from their perspectives... #psychosis #depression #bipolarclub #bipolar #bpd	• Informational support<br>○ Experiential informational support<br>○ Objective informational support<br>• Self-expression support		• Social awareness support<br>○ Perceptive awareness support

	Hey everybody, my pen name is… One day I'll have the courage to talk under my real name freely. Unfortunately, my work doesn't accept mental health struggles, so I use a pen name. I look forward to connecting with those in the #bipolarclub	• Informational support<br>○ Experiential informational support<br>• Self-expression support<br>• Network support		• Social awareness support<br>○ Perceptive awareness support<br>○ Destigmatization support
	A recommended post on my blog about "Navigating stigma with a mental illness"... #mentalillness #stigma #bipolar #bipolarclub	• Informational support<br>○ Experiential informational support<br>○ Objective informational support		• Social awareness support<br>○ Destigmatization support
Clarifying misconceptions about mental health conditions and fighting stigma	@AstrologyAccount Stop this! Making rude little posts comparing bipolar to astrology is despicable and stigmatizing...especially what is written does not describe bipolar disorder at all. Read something. #bipolarclub	• Informational support<br>○ Objective informational support		• Social awareness support<br>○ Perceptive awareness support<br>○ Destigmatization support
	Another side of #bipolardisorder, we don't have more than one personality and it isn't just mood swings. I hope this video reaches somebody who needs to understand this better, love you guys... #MentalHealthAwareness #bipolarclub	• Informational support<br>○ Experiential informational support<br>○ Objective informational support<br>• Self-expression support<br>• Emotional support		• Social awareness support<br>○ Perceptive awareness support
Groups and events for mental health support and awareness	Hey #bipolarclub! Join us today in our Twitter Space #DBTclub!... Topic: Healthy Relationships… Hosts: @CommunityCrewMember1… @CommunityCrewMember2… @CommunityCrewMember3… #bipolar #mentalhealth	• Informational support<br>○ Objective informational support<br>• Network support<br>• Tangible support		

	Good morning, #bipolarclub! Check out the recording of our Twitter Space #DBTclub if you missed it on Sunday... Learn how to be assertive in your relationships!... Hosts: @CommunityCrewMember1... @CommunityCrewMember2… @CommunityCrewMember3… #bipolar	• Informational support<br>○ Objective informational support<br>• Network support<br>• Tangible support		
	Making time for Black mental health event and save the date!... Event Date:… 👩🏾 Meet real Black therapists... A @ThinkTenacity event to freely access Black Therapists… 💻 Group support via Zoom… #Time2TalkSupport #bipolarclub #BlackWellness	• Informational support<br>○ Objective informational support<br>• Network support<br>• Tangible support<br><br>➢ *Support individuals from underserved and marginalized populations (Black population)*		
	Join us at our 2nd annual holiday music room and have some fun with us 🥳 🎉... #bipolar #bipolarclub #bipolarmusic	• Informational support<br>○ Objective informational support<br>• Network support<br>• Tangible support		
	I support proudly @samaritans @CLSamaritans this #BrewMonday…at...332...Road... Don't forget to remind everyone to reach out for a cuppa and a catch-up with the people you care about…#Listen #support #bluemonday #JanuaryBlues #DepressionIsReal #MentalHealthSupport	• Informational support<br>○ Objective informational support<br>• Network support<br>• Tangible support		• Social awareness support<br>○ Perceptive awareness support

	#MentalHealthMatters #MentalHealthAwareness #bipolarclub			
	Do you have any interest in a support club on Skype or like? #bipolarclub	• Informational support<br>○ Objective informational support<br>• Network support<br>• Tangible support		
Marketing mental health awareness products	My wristband designs of BIPOLAR :): AWARNESS are made and ready to buy on my Etsy store shortly. This is my first design and I'll make a bunch more if they're popular...what do you think? #bipolar #bipolarawareness #bipolarclub	• Informational support<br>○ Objective informational support<br>• Network support		• Social awareness support<br>○ Perceptive awareness support
**Behavioral coaching and motivational dialogue**				
Behavioral coaching tips and experiences	This morning, I tried out radical acceptance. I chose to accept that I would likely feel anxious instead of wishing I didn't feel anxious upon waking, and I used affirmations and breathing exercises to get through it. #bipolarclub	• Informational support<br>○ Experiential informational support<br>• Self-expression support		
	@CommunityMember Having self-control is good for you. #bipolarclub	• Informational support<br>○ Objective informational support<br>• Emotional support		
	Good morning, #bipolarclub! 🌞 Learn the 8 ways of how to stop being a chronic people pleaser!... #bipolar #bipolardisorder	• Informational support<br>○ Objective informational support		• Social awareness support<br>○ Perceptive awareness support

		• Emotional support		
Motivational dialogue	New 2023... Dear Me. 🌥️ Keep in mind every morning you're beautiful. 📖 I will track daily my bipolar moods. ❌ I won't put pressure on myself for 2023. 🤗 Never alone struggling with #bipolarLIFE... For SUPPORT @BipolarClubDx @BipolarUK @IntlBipolar #bipolarclub	• Informational support<br>○ Experiential informational support<br>○ Objective informational support<br>• Self-expression support<br>• Network support<br>• Emotional support<br>• Esteem support		
	When all I felt like doing was crying and calling in sick, I washed my hair and made it into work today. Gonna consider it a win… #bipolarclub #depression	• Informational support<br>○ Experiential informational support<br>• Self-expression support<br>• Emotional support		
	I can see a bit of light. No matter how small, I can see some light. ✨ Getting up and brushing myself off, with the support of friends, family, and the grace of God. One day at a time. #bipolar #bipolarclub	• Informational support<br>○ Experiential informational support<br>• Self-expression support<br>• Emotional support	• Family awareness support<br>• Friend awareness support	
**Personal feelings, thoughts, experiences, and reflections**				
Personal feelings, thoughts, and reflections	If I wasn't Muslim and suicide wasn't a sin, I won't probably be here anymore. #bipolarclub	• Informational support<br>○ Experiential informational support		

		• Self-expression support		
	How can everything smash in a night, I’m looking at suicide right now. #bipolarclub	• Informational support<br>○ Experiential informational support<br>• Self-expression support		
	So, at 4 am I get on the app and scroll through your stories. I do really care. But I couldn’t respond. So that’s me, despondent, discouraged, hurting (a bit), worried, and scared. Ending now this #vent. Good morning. #bipolarclub @BipolarClubDx #depression	• Informational support<br>○ Experiential informational support<br>• Self-expression support<br>• Network support<br>• Emotional support		
	When you don’t have any idea of what makes you happy, you feel even more depressed. I lay here alone, just thinking about my life, and I feel disappointed. I don’t feel right about things and I just want things to improve for me 😟 I wish I could get out of this funk #bipolarclub	• Informational support<br>○ Experiential informational support<br>• Self-expression support		
	From time to time, I think about people who don’t want to be with me because of me being bipolar, and it leads me to be sad at first, but then I feel like, well, everyone has a short life, they have the right to spend it with a normal person... #bipolarclub	• Informational support<br>○ Experiential informational support<br>• Self-expression support		• Social awareness support<br>○ Perceptive awareness support<br>○ Destigmatization support
	So grateful for the people who stand by us. It helps. #bipolarlife #bipolarclub	• Informational support<br>○ Experiential informational support<br>• Self-expression support		• Social awareness support<br>○ Perceptive awareness support

Personal negative experiences and coping struggles	Why my dad and his wife do still treat me as if I'm incapable of making a decision myself and I'm almost 57!!! Is that down to my #bipolardisorder #bipolarclub?	• Informational support<br>○ Experiential informational support<br>• Self-expression support<br>• Network support	• Family awareness support	
	I've just been re-traumatized by my family a week ago… Anyway, New Year, new me… ✌️ #CPTSD #trauma #toxicfamily #bipolarclub #HappyNewYear2023 #HappyHolidays	• Informational support<br>○ Experiential informational support<br>• Self-expression support<br>• Emotional support	• Family awareness support	
	Reading Emotionally Immature Parents book. Are your parents emotionally immature? How do you deal with it if so? #bipolarclub #EmotionalIntelligence	• Informational support<br>○ Experiential informational support<br>○ Objective informational support<br>• Network support	• Family awareness support	
	I keep having recognition more and more about the absolute hell of my childhood and I find it hard to find a way for some heavy feelings of anger. #bipolarclub	• Informational support<br>○ Experiential informational support<br>• Self-expression support<br><br>➢ *Support individuals from underserved and marginalized populations (children)*		
Hopes and prayers to cope with mental health conditions	"With great power comes great responsibility"… I wish I had a different life and was never hurt. I wish I could be normal every day as I'm not. Through	• Informational support<br>○ Experiential informational support		

	great suffering, real love and empathy come… #suicidal #bipolarclub	• Self-expression support<br>• Emotional support		
	The crash is the worst part of hypomania. And right before Christmas, too. Again! God, how I cope with this feeling. #bipolarclub	• Informational support<br>○ Experiential informational support<br>• Self-expression support		
